# Electroless Deposition and Nanolithography Can Control the Formation of Materials at the Nano-Scale for Plasmonic Applications

**DOI:** 10.3390/s140406056

**Published:** 2014-03-27

**Authors:** Maria Laura Coluccio, Francesco Gentile, Marco Francardi, Gerardo Perozziello, Natalia Malara, Patrizio Candeloro, Enzo Di Fabrizio

**Affiliations:** 1 Department of Experimental and Clinical Medicine, University Magna Graecia of Catanzaro, Catanzaro 88100, Italy; E-Mails: mlcoluccio@gmail.com (M.L.C.); gerardo.perozziello@unicz.it (G.P.); natalia.malara@iit.it (N.M.); patrizio.candeloro@unicz.it (P.C.); 2 Istituto Italiano di Tecnologia, Genova 16163, Italy; 3 Department of Physical Sciences and Engineering, King Abdullah University of Science and Technology (KAUST), Thuwal 23955-6900, Saudi Arabia; E-Mail: enzo.difabrizio@kaust.edu.sa

**Keywords:** metal nanoparticles, superclusters, electroless deposition, DLA, nanoscale systems, nano-optics and photonics, SERS

## Abstract

The new revolution in materials science is being driven by our ability to manipulate matter at the molecular level to create structures with novel functions and properties. The aim of this paper is to explore new strategies to obtain plasmonic metal nanostructures through the combination of a top down method, that is electron beam lithography, and a bottom up technique, that is the chemical electroless deposition. This technique allows a tight control over the shape and size of bi- and three-dimensional metal patterns at the nano scale. The resulting nanostructures can be used as constituents of Surface Enhanced Raman Spectroscopy (SERS) substrates, where the electromagnetic field is strongly amplified. Our results indicate that, in electroless growth, high quality metal nanostructures with sizes below 50 nm may be easily obtained. These findings were explained within the framework of a diffusion limited aggregation (DLA) model, that is a simulation model that makes it possible to decipher, at an atomic level, the rules governing the evolution of the growth front; moreover, we give a description of the physical mechanisms of growth at a basic level. In the discussion, we show how these findings can be utilized to fabricate dimers of silver nanospheres where the size and shape of those spheres is controlled with extreme precision and can be used for very large area SERS substrates and nano-optics, for single molecule detection.

## 1. Introduction

The discovery and understanding of fundamental principles on how nano scale systems of building blocks assemble, enables the engineering of nanomaterials with new properties in a variety of fields ranging from mechanics and photonics, to biology and medicine [1–8]. The behavior of similar systems is very often governed by scale dependent equations, where the discrete nature of these is connected to the geometrical and multi-scale character of the domain in which the energy exchanges occur [9]. Pure geometry and competition between surfaces and volumes are responsible for non-conventional, sometimes surprising, properties of nanodevices compared to their macroscopic counterparts [3,10]. In this view, metal nanomaterials promise a changing the way in which biosensing and molecular diagnosis are practiced, in that they feature certain clear advantages over conventional, continuous, macro scale materials [2,11].

Some of the attributes shared by metal nanomaterials are the ability of interacting with light [12], small sizes, at which much of biology occurs [13], a high degree of bio-compatibility [14,15], not complicated procedures for bio-functionalization [16,17] and elevated surface to volume ratios [18,19]. The small sizes in play and, more importantly than size, the shape, surface finish and internal structure of these nanosystems are responsible for the cited properties [20–24].

A wide variety of metal nanostructures is reported in literature, with shapes ranging from simple spheres, to more complex and sometimes exotic architectures, which include metal nanospheres [25,26], metallic films, [27,28] nanovoids, nanoshells, nanorods, nanorings and nanocubes [29–31]. The majority of these systems are assembled using the Langmuir Blodgett (LB) technique, where 2D super-lattices of colloidal structures are formed by pressure-induced organization at an air/water interface [32]. Like in the general case of colloidal synthesis, the nucleation process is critical for obtaining shaped metal nanoparticles, where shape control of metal nanocrystals can be carried out via either homogeneous [33] or heterogeneous [34] nucleation. In few cases, the self-assembly of supra-molecular structures is directed by either changing the energy or entropy landscapes, using templates or applying external fields [29].

**Figure 1.**
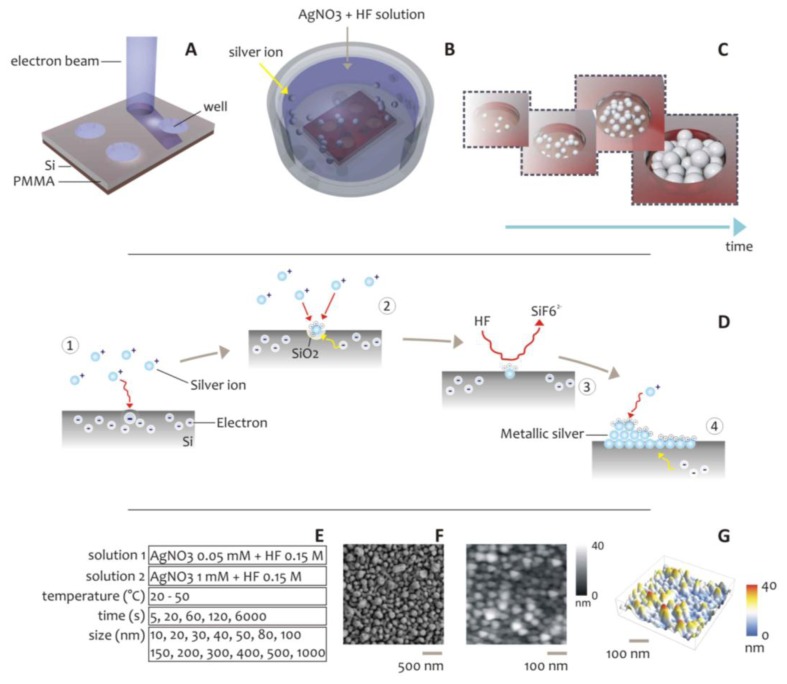
Scheme of the Electroless deposition process of silver nanoparticles aggregates. High resolution Electron Beam Lithography was used to create patterns on a silicon substrate (**A**). Those patterns were therefore exposed to a solution of AgNO_3_ and HF (**B**). Super-clusters of silver nanoparticles are obtained, where the form and size of those clusters is a function of the deposition time (**C**). In (**D**) we report the chemical reaction: Ag^+^ ions in the close proximity of the silicon surface capture electrons from the valence band of Si, a redox reaction between Ag^+^ and Si is initiated (D.1); Ag^+^ ions are reduced and deposited as metals while the silicon surface is oxidized into SiO_2_ (D.2); the redox reaction involves hydrofluoric acid, which induces the etching of SiO_2_ and the dissolution of SiF_2_^−^ (D.3). The Ag nuclei ttract electrons from bulk silicon, become charged negatively and, in a cascade effect, function as a catalytic surface for the reduction of further Ag^+^ ions (D.4). The table in (**E**) recapitulates the concentration of silver ions in solution, the size of lithographed patterns, the temperature and time of deposition, used in the electroless process. SEM (**F**) and AFM (**G**) images of a typical electroless grown silver nano-grains pattern.

As an alternative to the above described methods, here we propose a scheme for the formation of metal structures at the nanoscale. In the method, silver nanoparticles aggregates are self-deposited within nanowell patterns created by Electron Beam Lithography on a silicon substrate (Figure 1). This technique combines: (i) top-down (patterning via EBL) and (ii) bottom-up (the self-deposition of silver nanoclusters) approaches to afford accurate control over the shape and size of bi- and three- dimensional metal patterns at the nano scale.

The combination of top down and bottom up fabrication techniques has been, and is currently being, pursued by different research groups for a variety of applications, including the fabrication of highly efficient SERS substrates. While the general approach of the presented paper is not completely new, the combination of EBL patterning with a specific deposition technique, that is electroless growth, is an original scheme. To cite a few examples, in [35], Gopinath and colleagues demonstrated the use of a combined top down and bottom up fabrication process to obtain multi-scale systems with improved Raman sensing capabilities, where the top-down part of the process is represented by EBL patterning, like in our work. Nevertheless, in the cited paper, the authors utilized electron-beam evaporation for depositing the final nanoparticles. Differently from the described method, electroless growth allows realization of full three-dimensional structures, that is nanospheres, in opposition to disc-like, 2 + 1 dimensional structures, like those that may be obtained using a planar evaporation process. In [36], Pinna and colleagues obtained nanocomposite thin films formed by mesoporous titania layers loaded with ceria nanoparticles exposing the titania matrix with hard X-rays, where the exposition to hard X-rays triggers the formation of crystalline cerium oxides within the pores inducing the *in situ* growth of nanoparticles. Differently from this, our method does not require hard-X ray lithography and related instrumentation, including costly synchrotron radiation. Instead, the growth is site selective, and takes place in a solution of silver nitrate and hydrofluoric acid, that are compounds easily found in a chemical lab. Moreover, in our paper, rather than focusing on specific applications of the technique, we attempt to provide an explanation of the fundamental mechanisms of electroless particle formation at the nanoscale, using a joint experimental, numerical and theoretical approach. Also, none of cited the papers have the resolution and the geometrical/structural control found in our case.

Electroless deposition is a technique in which metal ions in solution can be reduced and deposited as metals using appropriate reducing agents, in presence of a catalyst that can accelerate the electroless reaction allowing for the oxidation of the reducing agent. In order to boost the transfer of electrons, both the metal ions and the reducing agent should be adsorbed onto the catalytic surface. While electroless deposition is a general process, we used here silicon as a plating substrate because it delivers the interesting ability to behave like a catalyst and reducing agent, simultaneously. This means that metal ions can be reduced as atoms on specific patterned sites of a silicon surface without the need of an external reducing agent. This property is determined by the presence of dangling bonds, which are deep-centre defects on the silicon surface, with a bound state that lies well below the conduction band as explained in [37]. On these basis, in the following we shall treat and describe the particular case of electroless deposition on a silicon surface.

In the described case, metal deposition takes place on the patterned substrate, where silver ions and silver dangling bonds react through a direct redox reaction to form the desired nanoparticles [31,38].

The scheme of the electroless chemical reaction is briefly summarized in Figure 1D. This is a particular case in which the reducing agent is the substrate itself, silicon, that through its dangling bonding, oxidizes and reduces silver ions to the metallic forms, as described by the following chemical reaction: [23,39–41]:

(1) $$4{\text{Ag}}^{+}+\text{Si}(\text{s})+6\text{HF}\to {4\text{Ag}}_{0}+{\text{H}}_{2}{\text{SiF}}_{6}+4{\text{H}}^{+}$$

which can be separated into two half-cell reactions, that are, the Si oxidation, as the anode:

(2) $$\text{Si}+2{\text{H}}_{2}\text{O}\to {\text{SiO}}_{2}{+4\text{H}}^{+}+4{\text{e}}^{-}$$

and the Ag reduction, at the cathode:

(3) $${\text{Ag}}^{+}+{\text{e}}^{-}\to {\text{Ag}}_{0}$$

In a first stage, the nanoparticle formation is dominated by the direct reaction of some silver ions with the silicon substrate, forming metallic nuclei. These Ag nuclei are strongly electronegative, and on account of this they attract other electrons from the silicon bulk becoming negatively charged; then new silver ions react with disposable electrons on silver grains, reducing to Ag_0_ and thus inducing the growth of the original Ag nuclei [22]. An autocatalytic mechanism is therefore induced, which continues also when all the silicon surface has been covered by silver, until electrons can be attracted from bulk silicon. In electroless deposition, the rate of the reactions is regulated by a balance between diffusion and kinetics, and these mechanisms are closely correlated [31]. Using this technique, a wide range of systems were realized, ranging from thin films, to sub-micrometric metallic structures and metal nanoparticles [38,42–45]. In [46], this concept was utilized for the realization of silver nanolenses, with single molecule detection capabilities, where the shape and size of those lenses was controlled at the nanometer level. In all the cited examples, the morphology of the aggregates depends on a variety of factors, including the *pH* of the solution, the temperature *T* of the system, the total time *t* of the process, the silver concentration *c* in solution and, more importantly, on the geometry of lithographed pattern. The effective area of silicon exposed to ions reaction affects the growth mechanism as a function of the availability of electrons. Despite this understanding, the mathematical comprehension of electroless deposition is still incomplete, and the process lacks predictive models, especially when combined with controlled geometries generated by nanolithography.

Preliminary studies on silver self-aggregation in nanodevices indicated that the morphology of metal nanoclusters depends on the pattern size. The authors of the present work demonstrated in [20] that, in nanosized structures, smaller patterns are preferable in forming continuous and compact ensembles of silver nanoparticles. Here, this concept was developed further. The dynamics of aggregation of metal nanoparticles was systematically examined as a function of the size and spacing of the lithographed pattern. Moreover, the concentration of silver ions in solution, the temperature of the system and the time of reaction, were also independently regulated.

Using different growth conditions reported in Figure 1E, silver nanoparticles aggregates were grown within well-defined patterns created by Electron Beam Lithography on silicon substrates (Figure 1A–C). Those nanoaggregates were therefore verified using direct SEM inspection (Figure 1F,G). The combined effects of the geometry and the growth conditions on size and distribution of the silver NPs, were analyzed. In doing so, we demonstrated a very high sensitivity of the roughness of the clusters of NPs to the variables of the process, and in particular to the pattern size and pattern spacing. Most importantly, we found a critical or limit diameter, below which the regime of deposition is linear, and isolated smooth spheres are obtained, to a region where instead the structures are fractured in a subset of minor blocks or particles. This critical diameter, for the particular subset of parameters used here, is about 50 nm, therefore, 50 nm is the preferred size for metal nanodevices for applications in photonics or fields where a precise and tight control of the quality of the grown structures is required.

These findings were explained within the framework of a numerical diffusion limited aggregation (DLA) model. DLA simulations, on linking bottom-up and top-down approaches, allowed us to address and resolve the multi-scale problem of electroless particle formation as a function of pattern size. Moreover, starting from basic principles, we tried to give a description of the physical mechanisms of growth at a fundamental level. In the discussion, we show how this concept can be utilized to fabricate dimers of metal nanoparticles which, conveniently positioned one respect to the other, can generate a controlled local electromagnetic (EM) field, crucial in enhanced spectroscopy and for few or single molecule detection.

DLA is a simulation method where particles stick together under a purely diffusive regime to form the intended structures. The resulting numerical structures may resemble or reproduce the real growths to a certain extent, but there will be always aspects regards to which the simulations will differ from the real structures, unless a molecular dynamics method is utilized, where the trajectories of atoms are determined by solving the Newton's equations of motion for a system of interacting particles, where forces between the particles and potential energy are defined by molecular mechanics force fields [47]. In that case, a perfect match between experiments and simulations is expected, at the cost though of massive computational times and resources, that prevents its utilization as a versatile simulation (predictive) tool. The convenience of using here a DLA model resides in its flexibility, ease of implementation, adeptness to represent real systems, and reduced simulation times. In the paper we elected the fractal dimension D_f_ as a key parameter for describing the numerical aggregates because it is a non-dimensional parameter, it may be derived on the basis on a solid and well assessed mathematical framework and, more importantly, starting from D_f_, the remaining parameters of the aggregates can be readily derived for direct comparison with the experiments. This is the case of the average grain size S, defined in the following of the article.

The paper is organized as follows: in the Experimental section we describe the methods utilized to fabricate the silver nanoclusters (Sections 2.1 and 2.2) and characterize the electroless growth (Section 2.3), and to conduct the spectroscopy measurement of the biological sample (Section 2.4). Next we describe in details the diffusion limited aggregation model we utilized to simulate the process and reproduce the results (Section 2.5) and the finite-difference time-domain (FDTD) methods we used to simulate the electromagnetic field around the grown structures (Section 2.6). In the Results, using experiments and computer simulations we show how to which extent the morphology of the nanograin aggregates is affected by the time and temperature of electroless deposition (Section 3.1) and, more importantly, by the pattern size (Section 3.2), moreover, using the classical nucleation theory, we try to explain the findings and the laws of growth at a fundamental level (Section 3.3). In the Discussion (Section 4), using computer simulations, we calculate the electromagnetic field enhancement in a dimer of silver nanoparticles with arbitrary size and spacing, and we demonstrate and discuss the ability of an ordered array of similar dimers to reveal molecules in a diluted solution. In the Conclusions (Section 5), we briefly review the results and propose possible directions for future work.

## 2. Experimental Section

The fabrication of aggregates of silver NPs occurred in two steps: (i) realization of micro- and nano- patterns by Electron Beam Lithography; (ii) electroless deposition of silver nano particles within the patterns. During e-beam lithography (Figure 1) the diameter *d* of nanostructures was varied between 10 and 1,000 nm. In depositing silver nanoparticles, the concentration *c* of the solution of AgNO_3_, the deposition time *t*, and the bath temperature *T*, were also varied in these intervals: *c* = (0.05, 1) mM, *t* = 5–6,000 s, *T* = (20, 50) °C; and different growth conditions were obtained. The combined effects of the geometry and the growth conditions, on size and distribution of the silver NPs, were therefore verified through direct SEM imaging. While the majority of conventional Scanning Electron Microscopes does not possess the necessary resolution to resolve details at the smaller scales that, in this case, are in the low nanometer range, here we used an SEM-FEI Nova 600 NanoLab system that permits the utilization of a particular configuration, namely the mode 2 configuration, whereby images can be magnified over 2,500,000 times and ultra-high resolution may be achieved (Figures S3 and S4).

De-ionized (D.I.) water (Milli-Q Direct 3, Millipore) was used for all experiments. Silver nitrate (AgNO_3_), hydrofluoric acid (HF) and benzenethiol (BT) were purchased from Sigma. All chemicals, unless mentioned otherwise, were of analytical grade and were used as received. p-Type (100) silicon wafers were thoroughly cleaned with acetone and ethanol to remove possible organic contaminants and then etched with a 20% wt HF solution (hydrofluoric acid 50% RPE ACS-ISO, Carlo Erba Reagents) to eliminate the superficial native oxide and obtain a passive hydrogenated surface (dangling bonding creation) [39]. Both the pretreatments were performed in a hot (40 °C) ultrasound bath.

### 2.1. Lithographic Process

Standard, p-type, 5–10 Ω/cm resistivity silicon wafers were used as substrates. An electronic sensitive resist, that is, ZEP for microstructures, and PMMA-A2 for nanostructures, was spin-coated for 60 s at 4,000 rpm (ZEP) and 5,000 rpm (PMMA) to obtain 70 (ZEP) and 50 (PMMA) nm thick layers. Prior the EBL exposure, the sample was pre-baked at 170 °C for 2 min to remove the solvent. The patterns of micro or nano-holes were exposed using a Crestec CABL-9000Ce-beam lithography system (Crestec Corporation, Tokyo, Japan), operating at 50 keV acceleration voltage. The samples were developed for 60 s in the ZED-N5 developer for the micro-structures realized in ZEP, and in isopropyl alchol at 4 °C for the nano-structures realized in PMMA.

### 2.2. Electroless Silver NPs Growth

The metal electroless deposition, in the patterned substrate, was realized by means of a redox reaction as described above. The driving force of the process is the potential difference *ΔE* between the two half-reactions. Nernst equation provides the relation between *ΔE* and the constant of equilibrium Ke of the global redox reaction:

(4) $$\Delta E=\frac{RT}{nF}\ln K_{e}$$

where *R* = 8.3143 J/mol·K is the universal gas constant, *T* is the absolute temperature of the system, *F* is the Faraday constant, *n*, is the number of electrons transferred in the reaction. Two different subsets of process parameters were used for depositing the metal ions into the nanolithographic trenches, that are recapitulated in Figure 1E and in the Supplementary Table S1, and which correspond to two different processes:

(i) a first process, where ΔE is higher, for clusters formation in microholes,
(ii) a second process, where *ΔE* is lower, for the realization of compact structures in nanoholes.

### 2.3. Sample SEM Characterization

SEM images of the samples were captured using a Dual Beam (SEM-FIB)—FEI Nova 600 NanoLab system (FEI, Hillsboro, OR, USA). During the acquisitions, the beam energy was set at 15 keV, and the corresponding electron current at 0.14 nA.

### 2.4. Raman Characterization of Benzenethiol Deposits

Microprobed Raman spectra were obtained using Renishaw inVia Raman microscope (Renishaw, Torino, Italy) at room temperature through a 100× objective of a Leica microscope (Leica Micro Systems, Wetzlar, Germany). The Raman spectra were excited by the 514.0 nm line of an Ar^+^ laser in backscattering geometry. The laser power was 0.18 mW with an integration time of 20 s. Mapping Raman measurements were carried out with the step size 0.5 μm in the x- and y-axis directions. For the measurements, we used the band centered at 1,580 cm^−1^ as a reference that corresponds to the C-C stretching mode in the molecule of benzenethiol.

### 2.5. Mathematical Description of Electroless Deposition

**Figure 2.**
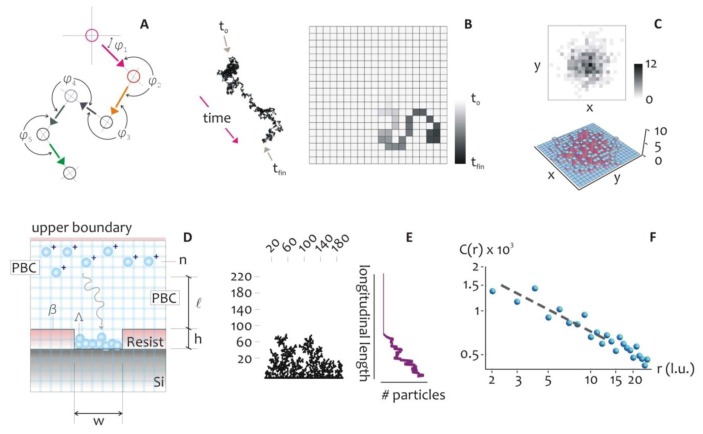
We used a Diffusion Limited Aggregation (DLA) model to simulate the electroless deposition of silver ions into supra-molecular structures. In the model, the trajectory of the particles can be described by a random walk, as to resemble a Brownian motion (**A**). The motion of a particle can be simulated in a regular lattice, where the smallest block of the lattice is called the lattice unit, that represents the spatial resolution of the simulation (that is, a pixel) (**B**). The distribution of displacements of a particle may be described using a Gaussian density function (**C**). This concept was used to simulate the deposition of metal ions in a patterned silicon substrate, (**D**), where a sufficiently large number n of particles is released in the system and let free to evolve. Using this scheme, an aggregate can be formed as in (**E**), where the multi branched arrangement of particles recalls the dendrite, fractal nature that electroless grown systems reveal under certain growth conditions. In (**F**), we report the pair correlation function of a similar numerical aggregate. A pair correlation function describes the change of information content of an aggregate as a function of change in scale and it is typically reported in a log log plot. In the diagram, the *y* axis represents the correlation function, while the *x* axis is the distance from the geometrical center of the aggregate expressed in lattice units (pixels). The pair correlation function is therefore related to the probability of finding the center of a particle a given distance from the center of another particle. For short distances, this is related to how the particles are packed together. On calculating the slope of this diagram, the fractal dimension of an aggregate can be derived.

The mechanism of metal growth was here reproduced under the assumption of a DLA process, where randomly displaced particles stick together to form the intended structures. This assumption holds is true when diffusion dominates over chemical reaction or, equivalently, when the kinetics of metal reduction is extremely fast (*t*_D_ ≫ *t*_K_), where *t_D_* and *t_K_* are the characteristic time scales of diffusion and chemical reaction. The model allows the understanding, at an atomic level, of the rules governing the evolution of the growth front, and to explore ways to tailor the morphology of the aggregates and their general characteristics [48,49]. In the model, the displacement of a metal ion, at any time, is arbitrary (Figure 2A), and thus the trajectory of the particles can be correctly described by a random walk, as in a Brownian motion.

At very short time scales, the motion of a particle is dominated by its inertia and its displacement will be linearly dependent on time, *Δx* = *vτ*, and this can be reproduced in a regular grid (Figure 2B) where particles are dislodged by the finite distance *Δx* in the time interval *τ*, that is the mean time between collisions. The instantaneous velocity of the particles, *v*, is maintained constant during *τ*, and it would depend solely upon the energy of the system. The distribution of probabilities of displacement of the Brownian particle itself is correctly best described by using a Gaussian density function, centered at on the origin of the walk: this is equivalent to say that the most probable position of a particle after a sufficiently large number of steps, is its original position (Figure 2C).

The root mean square (rms) distance of the walk is linearly proportional to time: It gives a measure of the extent of spread of the particle ensemble as described in [50]:

(5) $$<r^{2}>=<x^{2}>+<y^{2}>=4Dt$$

where *x*, *y*, and *r* are the Cartesian coordinates in the plane, while the celebrated Stokes-Einstein equation may be used to derive the diffusion coefficient *D* [3]:

(6) $$D=\frac{k_{b}T}{6\pi \mu a}$$

In Equation (6), *k_b_* is the Boltzmann constant, *T* the absolute temperature of the system, *μ* the viscosity of the medium, and *a* is the diameter of a particle with mass *m. D* can alternatively be expressed in terms of *Δx* and *τ* as:

(7) $$D=\frac{\Delta x^{2}}{2\tau }=\frac{{\left(\nu \tau \right)}^{2}}{2\tau }=\frac{k_{b}T}{2m}$$

where the kinetic theory of gases has been used for obtaining the right hand term of Equation (7) [50]. The variables utilized for the present configuration are recapitulated in Supplementary Table S2, where, in addition, we justify the application of the kinetic theory of gases to the diffusion of silver ions in a solution, as in the present model. Consider, for ease of visualization, the scheme in Figure 2D, where the diffusion and aggregation of metal ions, and their aggregation towards complex aggregates, is reproduced.

At a distance *l* from the well, *n* particles are released simultaneously in the system, where *n* at *t = 0* should be chosen with care to reproduce the initial concentration of silver ions. At any iteration the particles move within a regular square pattern of cells (the lattice) by one lattice unit (*l.u.*), and thus the mean path length *Δx* = *1* (l.u.). The lattice unit is the smallest block of the lattice and represents the shortest displacement that we can reproduce on the lattice. Equivalently, it is the spatial resolution of the simulation (that is, a pixel). At the left and right boundaries, that are, the walls of the well, periodic boundary conditions (PBC) are imposed, and when an individual particle crosses one of it, instantaneously reappears on the opposite face with the same velocity and *y* position. At the upper boundary, a bouncing condition is imposed, whereby the particles which collide with the wall would rebound downward. At the lower border of the system, the seed *Λ* represents the fresh silicon substrate exposed to growth: its length *w* may be adjusted according to the problem at study. *Λ* is thus a line of nucleation sites. The structure of the aggregate *β(t)*, would depend upon this initial seed, also notice that, at the initial time of diffusion, *β* coincides with *Λ*, being *β(0)* = *Λ*. When a particle migrates to touch *Λ*, it stops and it is incorporated by *Λ*, then the size of the aggregate is augmented by one unit. Simultaneously, a new particle is instantaneously created and randomly positioned in the upper region of the domain, therefore the total number of particles *n* is kept constant during the process. After a certain number of repetitions, an aggregate is formed as in Figure 2E, where the multi branched arrangement of particles recalls the dendrite, fractal nature that electroless growth reveals under certain conditions.

As regarding the simulation stop conditions, in the paper we investigated two different configurations, that are: (i) a varying pattern size, where the deposition time is maintained constant; and (ii) a constant patterns size, where instead the deposition time is varied. In case (i), the simulations were halted after a number of iterations N_i_ = O (2.5 × 10^11^), that according to the rules of conversion in the Supplementary Table S2, corresponds to an hypothetic true time of *t* = 20 s. In case (ii), the number of iterations was also linearly varied between the limits N_i-initial_ = 6.25 × 10^10^ and N_i-finale_ = 7.5 × 10^13^. The effects of the variation of temperature (from T_1_ = 293 K to T_2_ = 323 K) were instead included in the model simply reducing the number of iterations of a factor ι = v_2_/v_1_ = (T_1_/T_2_)^1/2^ = 0.95, according to the formula v = (K_b_ × T/m)^1/2^ reported in the Supplementary Section S2.

The structure of clusters of occupied lattice sites exhibit geometric scaling relationships which are characteristic of fractals and can be used to estimate an effective fractal dimensionality *D_f_* which has a value of about *D_f_* = 5/3∼1.667. The fractal dimension is a parameter that can be used to describe intimately the topography of a variety of systems, especially at the nano-scales [8]. A fractal dimension is an index for characterizing patterns by quantifying their complexity as a ratio of the change in detail to the change in scale [51]. When describing objects over multiple scales, it gives a measure of the complexity of those objects. Unlike topological dimensions, the fractal index can take non integer values, indicating that a set fills its space qualitatively and quantitatively differently from an ordinary geometrical set. In this case, a value of *D_f_* = 1.667 indicates that those aggregates are far more complex than a line, being distributed in the plane as they resemble a 1 to a 2 dimensional manifold. In comparison, the Sierpinski triangle has a fractal dimension of 1.585 [52]. The concept of fractal dimension, and the techniques needed to derive it, are recapitulated in [8,52,53], and are recalled in the Supplementary Section S3.

The importance of *D_f_* resides in the fact that it is used in the definition of certain properties which describe the deposit. As for instance, the mean cluster size *S* scales with the total number of deposited particles *N* in the aggregate as [54,55]:

(8) $$S∼N^{D_{f}/(D_{f}-1)}$$

thus, given *N*, that can be easily calculated, the mean cluster size would be readily derived. In the following, *S* is determined for either numerical DLA simulated aggregates, and experimentally grown electroless particles assemblies, as a function of the pattern size. These results were therefore compared and considered for deducing certain novel physical laws that would rule out the formation of metal particle clusters at the nano-scales.

A comprehensive list of parameters used for the simulations are recapitulated in Supplementary Section S2. In the paper, we used a classical DLA model to recover the experimental data. The model speculates that, if a diffusing atom sticks to the aggregate where it hits the aggregate, then fractal geometries will be formed. It neglects mechanisms such as (i) Adatom Diffusion on Terraces and Nucleation of Islands or (ii) Diffusion Along Island Edges, a detailed description of which is instead included in [49]. Therefore, the introduced model is a simplified hit and stick DLA (that is sometimes called, after Zhang *et al*. [49] regime I of fractal like growth, with zero local relaxation), where it is assumed that surface diffusion is much slower that the diffusion of silver ions in the bulk solution (or, alternatively, that surface diffusion is hindered by the large barrier between the sites).

DLA may be improved to include the effect of diffusion on the surface, simply decreasing the probability of sticking upon contact, that in the present configuration is fixed as *p* = 1 (that is, a deterministic process). In doing so, we would obtain and extended fractal growth (or regime II of DLA), where (differently from the regime I) the thickness *b* of the branches in a fractal set would be well larger than one atom (and thus, *b* > 1). In our model, *p* is an explicit, accessible variable, that was voluntarily set as *p* = 1 for computational convenience. Nevertheless, it may be arbitrarily changed to assume any real value between 0 and 1, to relax the constraint of a zero local relaxation (in this new regime II, an atom arriving at the edge of an island would have additional time to relax to a more favorable site before it is pinned in the position by the arrival of another atom). This more sophisticated version of DLA model would lead to more compact aggregates of atoms, compared to the fractal, strongly anisotropic numerical clusters that are shown in the present paper.

Despite this, the basic DLA model we used here revealed itself effective in capturing the basic mechanism of particle growth as a function of pattern size, as largely demonstrated in the results presented in the paper. Any further evaluation of the model can possibly lead to a more accurate description of the evolution of the growth front over time. Nevertheless, this understanding goes beyond the purpose of the present paper, and is left for future work.

Also notice how, while the electroless growth in the real world takes place in a three dimensional space, the DLA model implemented here would instead reproduce bi-dimensional systems. This strategy allows one to reduce dramatically the time needed for the simulations, still maintaining the capability to gain physical insight into the mechanisms of metal deposition at the smaller scale. While the use of a two-dimensional model is motivated by its simplicity and relative computational tractability, the electroless growth at the nanoscale is nearly a two-dimensional process, in that we are considering axial-symmetric channels where the transversal length is comparable to the dimension of the diffusing molecule. The transport can be therefore described in terms of the sole longitudinal (that is, perpendicular to the silicon substrate) and lateral (that is, parallel to the silicon substrate) coordinates, and this is a typical axial symmetric configuration. A combination of particle-wall hydrodynamic interactions and steric restrictions is responsible for this simplified representation of the problem thus disregarding any extra dimension [56].

Assuming the axial symmetry hypothesis as true, the average grain size in a similar 2-dimensional system, that is, the mean diameter of a cluster, would be the same found in the corresponding 3-dimensional system. In consideration of all this, we retain that the DLA 2D model, implemented in the present paper, is a reliable description of generic electroless growth phenomena.

Notice that, using a similar approach, we may use the fractal dimension of a simulated bidimensional set without further corrections, and this number is correctly comprised between 1 and 2 (that is, strictly smaller than the Euclidean dimension of a surface). If the axial symmetry hypothesis would break down, one may still use a bidimensional model, where the simulated 2D fractal set can be regarded as the projection of a compact fractal set in a 3D space into a lower dimension. In this case, with [57,58], one may asses the fractal dimension of the 3-dimensional aggregate simply as D_f_^3D^ = D_f_^2D^ + 1.

Deviations between the experiments and the simulations could arise because of the differences in the extent of particle spread with time, that in a three dimensional frame is proportional to (6 × D)^1/2^ [50], while in a bidimensional space is proportional to (4 × D)^1/2^. Therefore, the correction factor (3/2)^1/2^ was considered in comparing the experimental results with the theory.

### 2.6. Simulating the EM Field of Silver Dimers NPs

The resonant response of dimers of silver NPs was modeled using Lumerical™, a commercially available finite difference time-domain (FDTD) simulation software. The system at study is composed of two silver [59] hemispheres with a diameter of 55 nm, placed at a distance of 5 nm. Perfectly Matched Layers (PML) Boundary Conditions (BC) were designed to absorb incident light with minimal reflections at the boundaries of the simulated region. The system of two dimers was reproduced at the center of the simulated region (Supplementary Section S4, Figure S1). The entire system is supported by bulk silicon [60]. An adaptive mesh refinement technique was used whereby a fine 1 nm mesh grid is used for discretizing the spheres, where more resolution is required, and a coarser 100 nm mesh grid, instead, for the remaining domain.

A broadband plane wave, with wavelengths ranging between 30 nm and 600 nm, is generated in the vertical, z-axis direction, with polarization along the x-axis of the dimer (Supplementary Section S4, Figure S1); even if in spectroscopy a single frequency (monochromatic) incident beam is utilized, using here a broadband wave is convenient to determine the wavelength for which the EM field is maximized for a specific material. The data were acquired over specific regions of the domain, or monitors, conveniently positioned in the region of interest. Two monitors were used for the analysis: (i) 2D monitor placed in the section *xz* plane; and (ii), 3D monitor conveniently positioned in the gap between the two hemispheres. In (i), we determined the optimal wavelength (λ = 320 nm) and the z position (*z* = 8 nm, for *x* = 0, *i.e.*, in the middle of the gap) that would induce the highest field intensity. On using (ii), for a fixed *z* = 8 nm and *n* = 320 nm, we determined the field intensity, which is reported in Figure 5, where it is projected in the *xy* and *yz* planes. Notice that the intensity of the electric field is enhanced about 10^3^ times. In Supplementary Section S4, the intensity of the electric field is reported for a different direction of polarization, the y direction (Figure S2). In this case, the enhancement of the electric field is vanishingly small because is polarization sensitive due to the mode coupling of dimers.

## 3. Results

### 3.1. The Effect of Temperature and Time of Deposition

Several experiments were carried out to understand the effect of temperature and deposition time on the overall growth. The diameter of the lithographed pattern was chosen equal to 1 μm, that is sufficiently large to have a statistically significant number of nanometric grains during the growth. The electroless deposition time *t* was varied, being: *t* = 5, 20, 50, 120, 6,000 s, while two different temperatures, *T_1_* = 20 °C and *T_2_* = 50 °C, were imposed for the analysis.

**Figure 3.**
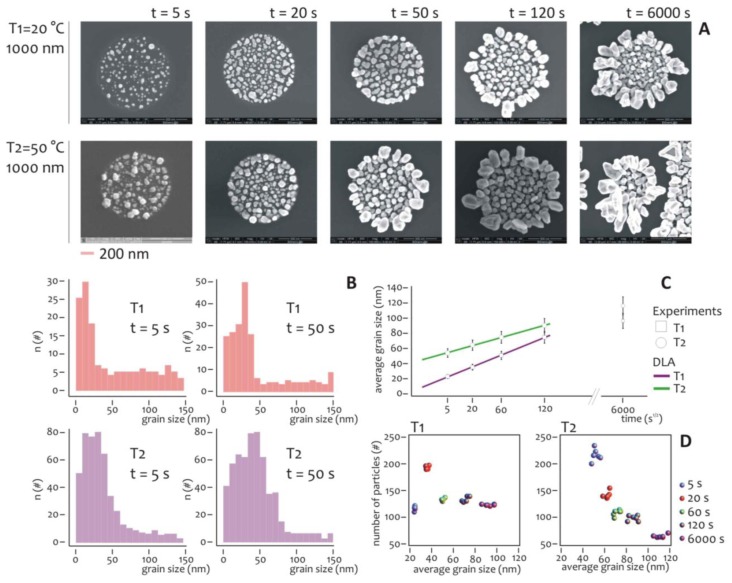
The effect of temperature and time of deposition on the characteristics of the aggregates of nano-particles. In (**A**), the SEM micrographs of the nano-particles aggregate, taken at low *T1* = 20 °C and high *T2* = 50 °C temperature, reveal the evolution of the growth front of silver with time (A). The cited SEM images can be conveniently processed and used to extract quantitative information of the clusters morphology. The average grain size distribution is reported in (**B**) for the limiting cases *T1* = 20 °C, *T2* = 50 °C, and for the time steps *t* = (5, 50) s. Complementary to this, the diagram in (**C**) reports the average grain size as a function of the square root of time, where a linear dependence between those variables is revealed, and this is consistent with the growth of particles in a diffusive regime. In (**D**), a scatter plot shows the number of particles as a function of particles size, for the time of the process of deposition varying between 5 and 6,000 s, for the fixed temperatures *T* = (20 °C, 50 °C). Incidentally notice how, at high temperature, the system is more sensitive to time, than at low temperature.

At each time step, SEM micrographs (Figure 3A) reveal the characteristics of the nano-grains assemblies and the differences between these for the (*T_1_*) and (*T_2_*) process temperatures. It can be observed that nano-grains are randomly distributed over the lithographed silicon surface, with a size that increases with time and that, on average, is smaller at *T_1_* compared to the *T_2_* temperature. Standard image analysis algorithms (Supplementary Section S5, in Figure S5a we show a representative SEM image of a cluster of nanoparticles, in Figure S5b, we show the size distribution derived from that image) were therefore utilized to gain quantitative details from the SEM images.

The grain size distribution was deduced per each image and reported for *t* = 5 s and *t* = 50 s, as in Figure 3B. The distributions of frequencies are skewed (that is, non-symmetric), whereby the tails on the right side are considerably longer relative to the left side, that is equivalent to say that, at the early stage of the process, smaller particles outnumber smaller ones. Considering that skewed data often occur due to lower or upper bounds on the data, two factors would explain the unbalance between small and large particles. The first is self-evident, that is, the dimension of the particles must be non-negative, and accordingly they would have a left/low bound of zero nm. The second origin of asymmetry is due to the critical nucleation size, that is the minimum size below which the particles are unstable and the probability of particle formation is almost equal to that of dissolution, in these conditions, the growth rate is much lower [61].

The nucleation size depends upon a variety of conditions including the temperature and concentration of metals ions: it plays a major role in the formation of nanoparticles aggregates especially in relation to the pattern size, as explained in the following of the paper. Interestingly, as the time of deposition increases or, equivalently, the grain size increases, the asymmetry is reduced, and the grains size events are arranged as to resemble a normal distribution. Notice that at the late stages of the process, the clusters of nanoparticles would have a characteristic length scale comparable with the dimension of the lithographic well, meaning that the border effects become important.

The diagram in Figure 3C reports the average grain size as a function of the square root of time for *T* = *T_1_* and *T* = *T_2_*. Both the traces are linear with t^1/2^ and this is consistent with the growth of particles in a diffusive regime [61]. As expected, at each deposition time interval, the nanoparticles grown at room temperature (*T_1_*), are smaller on average than those grown at higher temperature (*T_2_*). This difference, nevertheless, decreases with time, and can be ascribed to a saturation effect. After 10 minutes of process, the grains fill the well and the structures are overgrown, especially at the interface of the resist. Under these conditions, the hypothesis of diffusion limited aggregation breaks down, and the dimension of the aggregates becomes unpredictable. We notice that this regime is not interesting in this paper because we aim at obtaining the control of the growth on the nanoscale.

The number and size of particles aggregates can be visualized in a scatter plot as in Figure 3D, and here the differences between the two temperatures become visible. In the growth at lower temperature (*T_1_*), after a peak at the initial deposition time (that is, 20 s), the number of grains abruptly falls down and remain almost constant over time. Differently from this, at high temperature (*T_2_*), the number of particles aggregates decreases steadily with time, while their size increases as reported in Figure 3C. Thus, at the initial time of aggregation the more probable pattern configuration is composed by a large number of small particles. This configuration changes with time. As particle size increases, those particles would interact and merge together to form a few larger clusters. This can be easily explained considering that at low temperature the energy of the system is also low, and thus the particles are less prone to blend. More importantly, the differences between the diagrams would suggest different strategies for the design of metal nanoparticles clusters, and a lower temperature process would be preferable if one wants to work at a quasi-constant number of clusters, independently on the cluster size.

### 3.2. The Effect of Pattern Size

**Figure 4.**
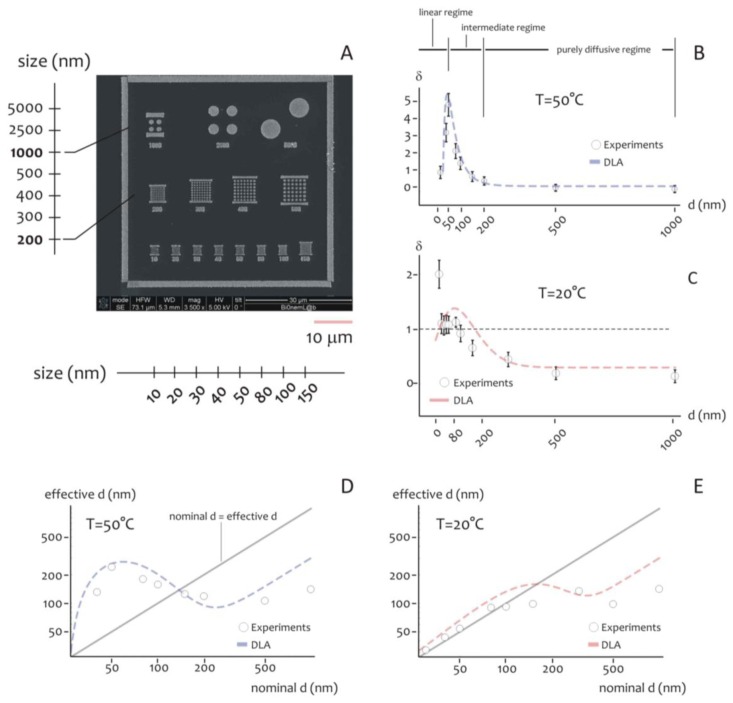
For analyzing the effect of pattern size on the characteristic of the nano-particles aggregates, we realized different lithographies, in which the size *d* of the patterns was varied from *d* = 10 nm, to *d* = 5,000 nm (**A**). The measured packing factor is reported in (**B**) as a function of pattern size *d* for fixed temperature (*T* = 50 °C) and time of deposition (*t* = 20 s). You may notice in the diagram three distinct regions, which correspond to different regimes of deposition. In (**C**), the packing factor is reported for *T* = 20 °C, the transition of the maximum towards larger values of *d*, can be explained making use of the classical nucleation theory (CNT). The information contained in (**B**,**C**) is organized differently as in (**D**,**E**), where the effective particle size obtained upon growth is reported as a function of the pattern size, that is, the nominal size of the nanoparticle. The diagram shows the effective particle diameter for a fixed pattern size, and has particular relevance for the nano-fabrication community.

As indicated above, the shape of the distribution of cluster size varies depending on the ratio between the average cluster size and the diameter of the lithographed pattern. This suggests that the size of the active area of deposition would rule the formation of the aggregates, especially at the nanoscale. Further experiments were therefore performed to verify this hypothesis. Circular patterns were produced with a diameter ranging from few to some hundreds of nm (Figure 4A), silver nanoparticles were therefore deposited within those patterns for 20 s at lower (*T_1_* = 20 °C) and higher (*T_2_* = 50 °C) temperature. The resulting structures were therefore analyzed using direct SEM imaging and, from those images, the average cluster size was derived as a function of pattern diameter (in a separate Supplementary Section S6, in Figures S3 and S4, we report a selection of SEM images of the silver nano-particles aggregates at the smaller nano-metric scales). In Figure 4B the ratio of the cluster size *S* to the nominal pattern diameter δ = *S/d* is reported for *d* varying from 10 to 1,000 nm, in this case the temperature of the system is set at *T* = *T_2_* (that is, higher temperature). Notice that δ gives a measure of the packing degree.

Specifically, if *δ* is smaller than one, the systems would be poorly compact or discontinuous, with a number of clusters n∼1/u^2^ greater than one. On the contrary, when h > 1, one single aggregate would uniformly fill the circular pattern with no, or minor, defects in its structure. In the particular case of δ > 1, the cluster size is larger than the pattern diameter, as indicated by specific images (Figures S6 and S7) reported in a separate Supplementary Section S7. In this case, the packing factor may be readily derived on measuring separately (a) the pattern size, that is the diameter of the exposed features in the EBL sensitive resist prior electroless growth, and (b) the nano-grains size obtained upon exposition to the electroless solution, and calculating the ratio between the two.

In the diagram of Figure 4B the three different regimes may be observed:

(i) In the linear regime, for low values of *d*, δ increases linearly with *d*, and it reaches a maximum when *d*∼50 nm, this the optimal value for our task where the shape of aggregates is almost spherical; we call this value *d_0_*;
(ii) In the intermediate regime, for moderate values of *d*, that is, from *d_0_* to approximately 2*d_0_*, δ is still larger than one, but it decreases with a certain power of *d*;
(iii) In the purely diffusive regime, for larger values of *d*, δ is smaller than one, meaning that the growth is discontinuous, and when the pattern size is sufficiently large (*d* > 3*d_0_*), δ reaches a constant state value: here the number of clusters per unit area would be almost constant. The reasons why the average grain size exhibits this behavior are not obvious, and are explained in a separated Results section, reported below. The cluster size, actually, is a function of the temperature of the system. At different on changing *T*, there is one should observe a change in the position of the maximum. This was verified at different temperatures as explained in the following. Figure 4C reports the plot of δ as a function of *d* at *T* = *T_1_* (that is, lower temperature). On comparing Figure 4B,C, please one can notice how the increase of the packing factor δ at high temperature (*T* = *T_2_*) is augmented with respect to the increase of δ at low temperature (*T* = *T_1_*), and this may be explained by the increased kinetics of the electroless reaction. Nevertheless, the three growth size regimes can still be observed at *T* = *T_1_*, where, most importantly, a maximum occurs again when *d* becomes equal to the critical nucleation diameter that, for the present configuration, is *d_c_* ∼ 80 nm, and, by comparing Figure 4B with Figure 4C, a shift is distinctly observable.

Nevertheless, the three regimes of growth are still clearly distinguishable also for *T* = *T1.* Here, as expected, the maximum occurs where *d* becomes equal to the critical nucleation diameter *d_c_* that, for the present configuration, is found as *d_c_* ∼ 80 nm.

The critical nucleation diameter is the minimal diameter that a nucleus should have for a nucleation process to proceed. The information contained in Figure 4B,C can be organize differently as in Figure 4D,E, where the effective cluster size is reported as a function of *d*, that is, the nominal size of the nanoparticle. For a reproducible fabrication, a preferable working region is where the deviations from the straight line with +1 slope is minimized. Close to the straight line, the fabricated value is close to the nominal *d* value. Figure 4D,E would suggest that working at *T_1_* = 20 °C is preferable for nominal pattern size ranging from 10 to about 100 nm.

Incidentally, in realizing the lithography, we utilized two different resists, for low (PMMA) and high resolution (ZEP) patterns. Although there have been observations regarding an important role of UV photoresists in determining the final morphology of the Ag dendrites, observing accumulation of dendrites at the photoresist edge, where a difference in stress is responsible for this [62], in analyzing the SEM micrographs of the Ag dendrites with attention to determine whether or not the UV photoresist affects the morphology of the dendrites at the edge, we did not observe the occurrence of this effect in our systems.

### 3.3. The Three Regimes of Particle Growth

Here, we propose a plausible explanation of the multiphase nature of particle growth observed in Figure 4B:

(i) *Linear regime*. The increase of cluster density with *d* is indeed counterintuitive. The classical nucleation theory (CNT) [61,63] predicts that, in a diffusive regime, the dimension *S* of a particle varies with the square root of time *t*:
  (9) $$S={\left(Dt+S_{0}\right)}^{1/2}$$
  In ordinary circumstances and at a fixed time, one would expect *d* to decrease with the diameter of the well. The diagram in Figure 4B suggests instead that certain geometric constrains limit the process of transport, deposition and aggregation of the ions to the clusters. These constrains depend on the lateral width of the well, and are progressively relaxed when *d* increases. Here, we argue that the growth mechanism of the nanoparticles is hindered for basically two reasons:
  The first, explains the reduced growth by the hindrance of molecular diffusivity in small nano channels. In pores, where the characteristic length scale is comparable to the dimension of the diffusing molecule, the transport of those molecules is reduced. A combination of particle-wall hydrodynamic interactions and steric restrictions are responsible for this reduction. A number of works were devoted to finding an analytical solution for this not purely diffusive phenomena, for applications ranging from drug delivery through nano-channels [64–66], to the study of static and dynamic properties of DNA in nano-pores, and DNA extension in nanotubes [67–69]. The diffusion hindrance factor *H* in confined geometries can be expressed as a power law of the particle to pore (pattern) diameter ratio, λ = *a/d*, and in the limit of vanishingly small λ, takes the form: [56,70]:
  (10) $$H(\lambda )=1+\frac{9}{8}\lambda \ln\lambda -1.539\lambda +1.2\lambda^{2}+o(\lambda^{2})$$
  Thus, larger pore geometries would induce a more pronounced diffusion, in accordance with the result of Figure 4B.
  The second mechanism that induces linear size dependent growth is, again, a lower bound in the nucleation formation of (nano) particles. The classical nucleation theory started from very basic physical principles some decades ago [61,63] and, despite a number of subtle, non-resolved questions, still appears to provide useful estimates in materials science. As for an example, it predicts the critical nucleation diameter *d_c_* in homogeneous nucleation, that occurs when, as in this case, the concentration of a solute in a solvent exceeds its equilibrium solubility:
  (11) $$\begin{array}{cc} d_{c}=\frac{4\gamma }{\Delta G_{\nu }}∼50nm & G_{\nu }=-\frac{K_{b}T}{\Omega \ln\left(\frac{\text{C}}{{\text{C}}_{0}}\right)} \end{array}$$
  In Equation (11) 1 =1.5 J/m^2^ is the surface energy per unit area of metallic silver [71], *ΔG_v_* the change of Gibbs free energy per unit volume of the solid phase, *Ω* = 1.16 × 10^−29^ m^3^ is the volume of a silver atom [72], *C/C_0_* = 10 is the super saturation of the actual to the equilibrium concentration of the system, *K_b_* is the Boltzmann constant and *T* = 323 *K* is the temperature of the system expressed in Kelvin degrees. Equation (11) is the result of a simple balance between surface energy and Gibbs free energy per unit volume, and it indicates the diameter below which the formation of a stable particle would be implausible. Notice that the critical diameter of nucleation *d_c_* coincides with the upper limit of our experimental linear regime of particle growth, *d_0_*, with excellent precision. The probability *p_0_* of a thermodynamic fluctuation associated to *d_c_* is:
  (12) $$p_{0}=e^{-\frac{16\pi \gamma }{3kT{\Delta {\text{G}}_{\text{v}}}^{2}}}$$
  that, using Equation (11), can be written as:
  (13) $$p_{0}=e^{-\frac{{d_{c}}^{2}\pi }{3kT\gamma }}$$
  In the present configuration, the size of the well *d* is smaller than the *d_c_* for a wide range of *d*. Assuming that, for any confined geometry with cross section d smaller than *d_c_*, the probability density function of nucleus formation is:
  (14) $$\xi (d)\delta d=p_{0}(1-e^{-d/d_{c}})\delta d$$
  the probability that a particle smaller than *d* would be generated can be therefore derived as the integral of *ξ(d)δd* over the considered range of diameters, that is:
  (15) $$p(d)=\int_{0}^{d}\xi (d)\delta d$$
  Equation (15) states that the formation of a stable particle depends upon the size *d* of the growth area. In consideration of these mechanisms, Equation (9) can be revised to include the contributions of (a) the molecular diffusion hindrance and (b) the size dependent particle nucleation probability:
  (16) $$s={\left(H(d)Dt+p(d)S_{0}\right)}^{1/2}$$
  the response of *S/d* in the restricted range of *d < d_0_* (between 10 nm and 50 nm) is linear, well in agreement with the experimental evidence and the DLA results.
(ii) *intermediate regime*. When the diameter *d* of the well becomes larger than *d_c_*, the nucleation of a new particle is no more restricted by geometric constrains. Moreover, the aspect ratio of the structure drops off and thus the diffusion coefficient is recovered to its original value. Under these conditions the average cluster size is constant at a given time (Equation (9)) and δ = *S/d* would decrease with *d*.
(iii) *purely diffusive regime*. When *d* is sufficiently large, *S* is small compared to *d*, and thus h is nearly zero. Notice that in this region of the diagram the number of clusters will always be larger than one, and the overall structure will be constituted by a number of different clusters or patches rather by one continuous block. To demonstrate this, we clothe the problem in a probabilistic form. At the initial deposition time, the nucleus size is *d_c_*. In the well there will be, therefore, *n* = (*d/d_c_*)^2^ particle seeds, with *n* ≫ *1*. We posit now the simplifying assumption that, as in a competition process, each nucleus has the same probability *g* = *1/n* to conquer the region around prevailing on the remaining nuclei. Accordingly, the probability that a nucleus does not prevail on the others, after a finite time, is *q* = *1* − *g*. Therefore, the probability that none of the nuclei will prevail on the others, is *u* = *q* × *n* = *(1* − *1/n)n*. By definition, *u* is also the probability of finding a uniform clusters size distribution. If *n* or, equivalently, *d*, is large, then *g*→0 and *u*→e^−1^, that means that *n* aggregates would be uniformly incorporated by the system.

## 4. Discussion

The control of the process of deposition and formation of metal nanoparticles aggregates can be important in optical devices, where the geometry plays an important role in determining the local electromagnetic field.

In particular, Surface Enhanced Raman Scattering (SERS) is an effect where the electromagnetic field is locally enhanced by the resonant interaction of light with the surface plasmons polaritons in the metal [73,74]. Surface plasmons polaritons (SPP) are collective oscillations of conduction electrons excited by an electromagnetic field. Through scattering, SPP, in turn, can generate an inverse process where light is radiated by the metal, both, in near and far field components. A molecule, adsorbed on the metal nano-cluster, would thus feel an increased field intensity, and the Raman spectroscopy signal would be accordingly amplified. And thus the ingredients for SERS are basically two:

(i) the features have to be fabricated on a length scale comparable with the λ/10 (λ is the electromagnetic wavelength),
(ii) the distance between the elementary nano structures has to be controlled on a length scale of about λ/100.

This two length scales are the reason why nanoplasmonics and nanofabrication are tightly connected.

While randomly rough surfaces still induce a sensible enhancement in the signal of the electric (E) field [11,75], regular geometries are the best building blocks for advanced optical devices, where a good theoretical control can be achieved for a desired optical response. For certain architectures, this response can be extremely strong, with |E|^4^ enhancement up to five orders of magnitude [21,76–82]. In the following, we will treat the case of a dimer. Notably, on the experimental side, the behavior of real dimers has been described by a limited number of works solely, the majority of which deals with colloidal particles, randomly dispersed in a plane [83]. The fabrication of nanowells and reproducible geometrical systems at the nanoscale, and the precise positioning of those systems in the space, has remained an elusive goal for a long time.

In the presented results, we demonstrated that for a 50 °C temperature and for dimensions smaller than about 50 nm, nearly spherical structure can be obtained, with a continuous smooth surface, and with a linear dependence of the growth on the diameter. This linear regime may be extended to about 80 nm on decreasing the temperature of the process to 20 °C. Nevertheless, for this configuration, the formation of the particle aggregates would be less sensitive on the variation of the diameter and the overall growth would be restricted. We gave an explanation to these observations.

**Figure 5.**
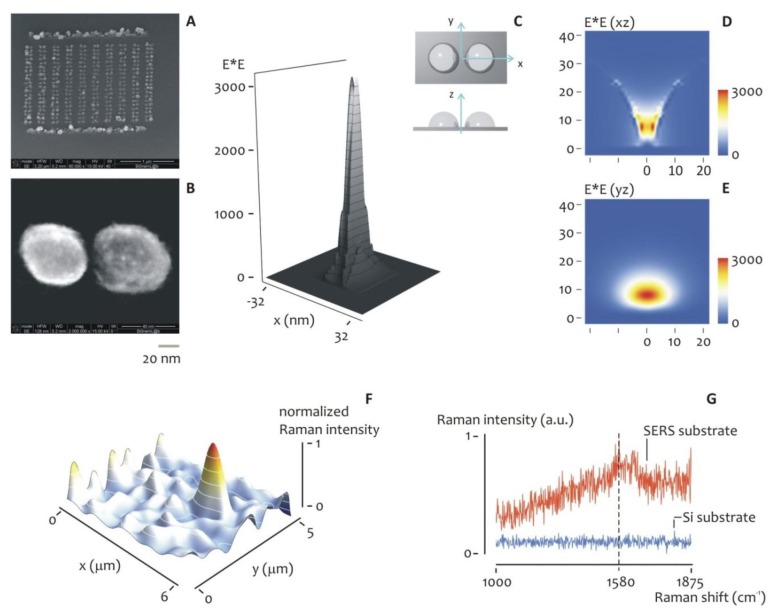
The laws of electroless particle deposition at the nano-scales, revealed in Figure 4, were utilized to fabricate dimers of silvers nano-particles. The low magnification SEM image in (**A**) show the ability of the method of realizing silver dimers of nano-particles on a large scale, where the size and spacing of those spheres is controlled with extreme precision, as revealed by the high magnification SEM image in (**B**). In (**C**), a 3d representation of the field intensity of the electromagnetic field in the plane of the substrate is reproduced, while in (**D**,**E**) the projection of such a field is reported in the *xz* and y*z* planes, where major details are shown, and where it can be noticed how the enhancement in the electrical filed intensity is as large as 10^3^. The detection capabilities of a system of similar dimers were therefore verified using, as a probe, benzenethiol (BT). A 3d Raman intensity map of the sample acquired over a region of interest large 5 × 6 microns is shown in (**G**). (**F**) shows two individual SERS spectra, acquired on the SERS (silver-dimers) substrate and simple silicon, respectively, where the difference in intensity between the two demonstrate the increased efficiency of the array of silver dimers as a SERS substrate.

Therefore, we verified the electroless capability of producing regular nanostructures even further. Specifically, we used this original comprehension of the nanoparticle formation mechanisms to fabricate arrays of dimers on the basis of a criterion of rational design. In finite domain time difference (FDTD) calculations for silver nanoparticles and dimers of silver nanoparticles in which the size of the nanoparticle size and spacing of the nanoparticles were varied over a significant range, we found that the dimers give maximum E-fields that are a factor of 10 larger than any compact monomer structure. Moreover, we observed a low sensitivity to local radius of curvature, but an elevated dependence on particle spacing, thereby doublets (or dimers) of nano particles with a diameter of 50 nm and a gap of 5 nm, would favor the formation of the most intense electric field. Notably, 50 nm is also the optimal diameter for an electroless deposition conducted at *T_1_* = 50 °C temperature. Considering these results, we fabricated a certain number of dimers as to reproduce this particular geometry (Figure 5).

Several SEM investigations of the dimers were done over different samples to assess uniformity and reproducibility. In Figure 5A, those silver nanoparticles are arrayed over large square areas of few micrometers per side. On a smaller scale (Figure 5B), the nano-structures exhibit a round, continuous profile with slight deviations from a perfect sphere. The positioning of these structures in the plane is controlled with e-beam nanolithography, with the particle-particle distance (gap) practically equal to the nominal value of 5 nm. The SEM images in Figure 5 reveal the ability of the electroless growth process to realize nano-particles aggregates, nano spheres or particulates with desired precision.

In Figure 5C, a 3D representation of the field intensity in the plane of the substrate is reproduced, while in Figure 5D,E the projection of such a field is reported in the *xz* and *yz* planes, where major details are shown, and where the electrical enhancement field reaches 10^3^.

The detection capabilities of a system of similar dimers were therefore verified using, as a probe, benzenthiol (BT); solutions were prepared containing BT molecules with a concentration as low as 10^−16^ M. We deposited 2 μL of this solution on the devices and let it evaporate. In doing so, few molecules were positioned partially on the ordered array of silver dimers and partially on simple silicon. Micro-Raman mapping measurements were therefore performed. Figure 5F shows a 3D Raman intensity map of the sample acquired over a region of interest large 5 × 6 microns. Figure 5G shows two individual SERS spectra, acquired on the SERS (silver-dimers) substrate and simple silicon, respectively, where the difference in intensity between the two demonstrate the increased efficiency of the array of silver dimers as a SERS substrate. Moreover, in a separate Supplementary Section S8 we report (i) the reference spectrum of benzenethiol (Figure S8A); (ii) the spectrum of benzenethiol measured using a monomer particles array compared to the Raman signal of the same solution acquired using a system of dimers (Figure S8B) and (iii) the Raman signal of the solvent of benzenethiol alone, that is, ethanol (Figure S9): these are the controls necessary to demonstrate that the signal observed in Figure 5F,G may be correctly attributed to BT and are not generated by the solvent or other contaminants.

Aside from this specific example, the electroless growth at the nanoscale, may furnish new basic tools for application to nanooptics and photonics, overcoming the stringent geometrical requirements of plasmonics nanostructures.

## 5. Conclusions

In this work, we demonstrated that a combination of *top down* nanolithography and a *bottom up* electroless deposition growth can bring about the fabrication of well controlled metallic nanostructures of deep interest in nanoplasmonics. Using e-beam lithography a local metal electroless deposition method was developed. SEM investigation showed that the growth depends upon a variety of parameters, including the growth time, the temperature of the system and the initial concentration of silver ions. Further, it was demonstrated that the characteristics of the structures is a function of the pattern size. In particular, the transition from small to large geometries is found to occur for a characteristic or critical diameter that, for a process temperature of 50 °C, is about 50 nm. This critical diameter depends, in turn, on the temperature of the system, and would augment for decreasing *T*.

Finally we gave a physical explanation of this behavior. Moreover, founded upon the DLA paradigm that, in the limit of very fast chemical reactions, diffusion is the sole driving force that regulates the dynamics of aggregation of NPs, we derived a model to substantiate these findings. The model reproduces the fundamental operating mechanism of silver electroless deposition in good agreement with the experimental observables, and can thus be used as a criterion for the rational design of metal structures at the nanoscale when regular shapes and sizes are necessary, as in nanophotonics. These results suggest working at small pattern sizes for obtaining isolated, uniform and reproducible metal nanospheres.

## Supplementary Material
