# Supplementary material for: Electroless Deposition and Nanolithography Can Control the Formation of Materials at the Nano-Scale for Plasmonic Applications
Source: Sensors (Basel). 2014 Mar 27;14(4):6056–83. doi: 10.3390/s140406056 (PMC4029638; doi:10.3390/s140406056)
Supplement: Supplementary file 1 [file sensors-14-06056-s001.pdf]

OPEN ACCESS

sensors

ISSN 1424-8220

www.mdpi.com/journal/sensors

Supplementary Information

Electroless Deposition and Nanolithography Can Control the Formation of Materials at the Nano-Scale for Plasmonic Applications. *Sensors* 2014, *14*, 6056-6083

Maria Laura Coluccio [1,†], Francesco Gentile [1,2,†,*], Marco Francardi [3], Gerardo Perozziello [1], Natalia Malara [1], Patrizio Candeloro [1] and Enzo Di Fabrizio [1,3]

[1] Department of Experimental and Clinical Medicine, University Magna Graecia of Catanzaro, Catanzaro 88100, Italy; E-Mails: mlcoluccio@gmail.com (M.L.C.); gerardo.perozziello@unicz.it (G.P.); natalia.malara@iit.it (N.M.); patrizio.candeloro@unicz.it (P.C.)

[2] Istituto Italiano di Tecnologia, Genova 16163, Italy

[3] Department of Physical Sciences and Engineering, King Abdullah University of Science and Technology (KAUST), Thuwal 23955-6900, Saudi Arabia; E-Mail: enzo.difabrizio@kaust.edu.sa

† These authors contributed equally to this work.

* Author to whom correspondence should be addressed; E-Mail: gentile@unicz.it; Tel.: +39-010-7178-1551.

S1. The Detailed Set of Values Used for the Electroless Deposition of Silver Grains

Table S1. The detailed set of values used for the electroless deposition of silver grains.

**Metal Species**	Silver (Ag)
**HF Concentration in Solution**	0.15 M
**Ag Concentration in Solution**	0.05 mM (size < 1,000 nm)<br>1 mM (size > 1,000 nm)
**Pattern Size (nm)**	10, 20, 30, 40, 50, 80, 100, 150, 200, 300, 400, 500, 1,000, 2,500, 5,000<br>(the deposition time is hold fixed = 20 s)
**Time of Deposition (s)**	5, 20, 50, 120, 6000<br>(the deposition time is hold fixed = 20 s)
**Temperature of Deposition (℃)**	20, 50

S2. The Parameters Used for the Diffusion Limited Aggregation (DLA) Simulations of Electroless Deposits

The variables utilized in the DLA simulations assume, for the considered case, prescribed values as recapitulated in the following Table S2.

Table S2. The physical constants used for the simulations.

Variable	Symbol	Units	Value	Source
Mass of a silver ion	m	[Kg]	17×10^{-25}	Reference [1]
Temperature of the system	T	[k]	323	-
Velocity of the ion	v	[m s^{-1}]	51	$\sqrt{k_b T/m}$
Viscosity of water at T = 323 K	μ	[Pa s]	0.3×10^{-3}	Reference [1]
Diffusion coefficient	D	[m^{2}s]	3.95×10^{-9}	$k_b T/6\pi\mu a$
Mean path length	Δx	[nm]	0.3	$4D/v$
Time interval	τ	[s]	$\sim 10^{-11}$	$\Delta x/v$

The meaning of the time parameter t above, is that of an estimate of a hypothetical true external time that a clock measures. The time interval ntcan be considered as the simplest basis, or subunit, of this absolute time. An interaction, that is, the dislodgement of a metal ion by a lattice unit, takes the time τ, and the system cannot see or sense any time shorter than τ. To this extent, the total time can be discretized in τ units. τ is not arbitrary, and instead depends on physically observable variables of the system, such as the temperature T, the mass m and diameter d of the dislodging ions, the viscosity of the medium.

Notice that, while the electroless growth in the real world takes place in a three dimensional space, the DLA model implemented here would instead reproduce bi-dimensional systems. This strategy allows one to dramatically reduce the duration of the simulations, while still maintaining the capability to gain physical insight into the mechanisms of metal deposition at the smaller scales. While the use of a two-dimensional model is motivated by its simplicity and relative computational tractability, the electroless growth at the nanoscales is nearly a two-dimensional process, in that we are considering axial-symmetric channels where the transversal length is comparable to the dimension of the diffusing molecule. The transport can be therefore described in terms of the sole longitudinal (that is, perpendicular to the silicon substrate) and lateral (that is, parallel to the silicon substrate) coordinates. A combination of particle-wall hydrodynamic interactions and steric restrictions is responsible for this simplified representation of the problems thus disregarding any extra dimension [2]. Moreover, in comparing the experiments to the theory, we used the ratio between the characteristic length scale of the aggregates to the dimension of the pattern, that is a non-dimensional parameter. In consideration of all this, we retain that the DLA 2D model, implemented in the present paper, is a reliable description of generic electroless growth phenomena.

Deviations between the experiments and the simulations could arise because of the differences in the extent of particle spread with time, that in a three dimensional frame is proportional to $(6D)^{1/2}$ [3], while in a bi-dimensional space is proportional to $(4D)^{1/2}$ (Equation (1)). Therefore, the correction factor $(3/2)^{1/2}$ was considered in comparing the experimental results with the theory.

As regarding the assumption that "At any cycle the particles move within a regular square pattern of cells by one lattice unit (l.u.)", made in the work: here we substantiate this hypothesis. Using as the average diameter of silver ion the value $a = 0.2 \times 10^{-9}$ m [1], from the celebrated Stokes-Einstein relation we may derive the diffusion coefficient of those ions as D = ($K_b \times T/6 \times \pi \times mu \times a$) = 3.95×10^{-9} (m^2 s), where K_b is the Boltzmann constant, T = 323 K the temperature of the system, mu = 0.3×10^{-3} Pa s the viscosity of water at T = 323 K. The velocity of silver ion, between a collision and another, is $v = (Kb \times T/m)^{1/2} = 51.1$ m/s, where $m = 17 \times 10^{-25}$ Kg is the mass of a silver ion [1]. Therefore, the mean path length can be derived as $\Delta x = 4D/v = 0.3$ nm, that is roughly the diameter of the ion. The assumption that "At any cycle the particles move within a regular square pattern of cells by one lattice unit (l.u.)" is equivalent to say that, in the time constant tau, each particle of size a travels a distance a, that is, exactly its size, and has been proved above. Incidentally, notice how the DLA model we utilized permits regulating at will the mean path length Δx of the dislodging ions, that can be arbitrarily fixed as an integer number of times the lattice unit, according to the problem in analysis (that is to say, the displacement of ions with an arbitrary size and mean path length, can be correctly reproduced in the grid).

As regarding the usage of a kinetic theory similar to that of rarefied gases, the kinetic theory of gases describes a gas as a large number of small particles (atoms or molecules), all of which are in constant, random Brownian motion. This has evident similarities with the metal ions in solution, where ions are transported by a purely diffusive process. Notice that diffusion, and the laws of diffusion, is a way to express an otherwise complex process in statistical form. In deriving the laws of diffusion, one makes explicit use [3] of the formula for the velocity of a particle between collisions, being $v = (K_b \times T/m)^{1/2}$, that is a direct result of the kinetic theory of gases.

The use of the mathematics of diffusion, as in the paper, is possible provided that the following assumptions hold true [3]: (a) The ensemble consists of very small particles. This smallness of their size is such that the total volume of the individual ions added up is negligible compared to the volume of solution. This is equivalent to stating that the average distance separating the ions is large compared to their size; (b) These particles have the same mass; (c) The number of ions is so large that statistical treatment can be applied; (d) These molecules are in constant, random, and rapid motion; (e) Except during collisions, the interactions among molecules are negligible. (That is, they exert no forces on one another). This also implies that the ions are treated as classical objects, and that the equations of motion of the ions are time-reversible.

Notice that all these requirement are met by the Ag^+ ions. Condition a is particularly important. Even considering the most concentrated solution (1 mM), one would have approximately 1,020 ions per liter of solution, that is equivalent to having roughly $10^{20}/3 = 5 \times 10^6$ ions aligned in a mono dimensional line with a length of 10 cm. In this case, the distance between ions would be 21 nm, that is more than two orders of magnitude larger than the Van der Waals size of a silver ion, that is ~0.2 nm [1].

S3. The Concept of Fractal Dimension, and Its Application to the Analysis of the DLA Simulated Aggregates

Fractals are mathematical objects that are too irregular to be described by conventional geometry. They all retain, to different extents, certain properties that may be reviewed as follows: (i) they reveal

details on arbitrarily small scales (fine structure); (ii) they can be generated (and thus described) by short algorithms (perhaps recursively); (iii) they exhibit a *fractal* dimension D_f strictly greater than the classical topological dimension [4–6]. The latter property reserves particular attention, in that it claims that a surface, under a fractal point of view, may have a dimension D_f even greater than 2, and the more D_f is close to 3, the more the fractal set *fills* the space it is embedded in. The fractal dimension of the simulated nanoparticles aggregates can be derived using certain mathematical procedures as described below:

The power spectrum of a fractal set. [6] A power spectrum (PS) can be associated to each of the DLA generated deposits. The PSdensity function:

$$C_{2D(q)=\frac{1}{(2\pi)^2}\left(\int\langle z(\chi)z(o)e^{-iq\chi}d\chi^2\rangle\right)} \tag{S1}$$

contains relevant information regarding the microstructure of the aggregates in that represents the energy content per each image. In (S1), q is the wave number, related to the characteristic wavelength λ as $q = 2\,\pi/\lambda$; $\chi = (x, y)$ is the planar coordinate; $z(\chi)$ is the surface profile measured from the average surface plane, defined as $<z>=0$; and the symbol $<\ldots>$ stands for ensemble averaging over a collection of different surfaces with identical statistical properties. Since the 2D power spectrum density introduced above is impractical for comparison purposes, a 1D power spectrum density can be conveniently extracted using the transformation:

$$C(q) = \frac{1}{\Gamma}\oint_{\Gamma} C_{2D}(q_x, q_y)dy = \frac{1}{2\pi}\int_0^{2\pi} C_{2D}(q\,cos\Psi, q\,sin\Psi)d\Psi \tag{S2}$$

where the polar variables $q = (q_x^2 + q_y^2)^{1/2}$, $\psi = \arctan(q_y/q_x)$, in the plane (x, y) of interest have been considered.

Deriving the fractal dimension from the power spectrum density function [6]. In the case of self-affine surfaces, for which a rescale in the planar coordinates $x \rightarrow bx$ and $y \rightarrow by$ is accompanied by a rescaling in the normal direction $z(b\chi) \rightarrow bHz(\chi)$, the power spectrum $C(q)$ takes the form:

$$C(q) = \frac{H}{2\pi}\left(\frac{h_0}{q_0}\right)^2\left(\frac{q}{q_0}\right)^{-2(H+1)} \tag{S3}$$

for $q > q_o$, where q_o is the lower cut-off wavenumber corresponding to an upper cut-off wavelength $\lambda_o = 2\pi/q_o$; and h_o is related to the rms roughness amplitude as $h_o = 2^{1/2}$ Rrms. A self-affine fractal surface can be consequently univocally identified by specifying the surface roughness (Rrms), the cut-off wavenumber q_o and the coefficient H, known as the Hurst coefficient. In a loglog plot, the power spectrum density appears as a line with a slope β for $q > q_o$ (see, as for an instance, Figure 2F in the main text). The slope β is related to the Hurst parameters as $\beta = 2(H + 1)$. The fractal dimension D of the surface can be derived from β or H as $D = (8 - \beta)/2$ or $D = 3 - H$. The fractal dimension D for a surface ranges from 2, representing a perfectly flat surface (Euclidean dimension of a surface), to 3, representing an extremely rough surface. For $D = 2.5$, the so-called Brownian surfaces are identified which have totally random and uncorrelated profiles.

Deriving the Power Spectrum from the pair correlation analysis of numerical DLA aggregates [5]. The power spectrum of a signal gives the distribution of the signal power among various frequencies. The power spectrum is the Fourier transform of the correlation function, and reveals information on

the correlation structure of the signal. For a discrete set of data, as those obtained through the DLA simulation described in the Section S2, the power spectrum coincides with the Fourier transform of the pair correlation of those data, $C(r)$.

The pair correlation function is related to the probability of finding the center of a particle a given distance from the center of another particle. For short distances, this is related to how the particles are packed together. For example, consider hard spheres, like marbles. The spheres can't overlap, so the closest distance two centers can be is equal to the diameter of the spheres. However, several spheres can be touching one sphere; then a few more can form a layer around them, and so on. Further away, these layers get more diffuse, and so for large distances, the probability of finding two spheres with a given separation is essentially constant. In that case, it's related to the density—a more dense system has more spheres, thus it's more likely to find two of them with a given distance. The pair correlation function $C(r)$ accounts for these factors by normalizing by the density; thus at large values of r it goes to 1, uniform probability. Here we provide an explanation of how $C(r)$ can be calculated: [A] Pick a value of dr [B]. Loop over all values of r that you care about: (1) Consider each particle you have in turn. Count all particles that are a distance between r and $r + dr$ away from the particle you're considering. You can think of this as all particles in a spherical shell surrounding the reference particle. The shell has a thickness dr. (2) Divide your total count by N, the number of reference particles you considered—probably the total number of particles in your data. (3) Divide this number by $4\pi r^2 dr$, the volume of the spherical shell (the surface area $4\pi r^2$, multiplied by the small thickness dr). This accounts for the fact that as r gets larger, for trivial reasons you find more particles with the given separation. (4) Divide this by the particle number density. This ensures that $C(r) = 1$ for data with no structure. In other words, if you just had an arbitrarily placed spherical shell of inner radius r and outer radius $r + dr$, you'd expect to find about ρ V particles inside, where ρ is the number density and V is the volume of that shell. In 2D, follow the algorithm as above but divide by *2yvr* dr instead of step 3 above.

S4. FDTD Simulations of the EM Field around Dimers of Silver NPs: The Effect of a Different Polarization Direction

The FDTD simulations reproduced a system of two silver hemispheres with a diameter of 55 nm, placed at a distance of 5 nm, at the center of the region as in the Figure S1A. A broadband plane wave, with wavelengths ranging between 300 and 600 nm, is generated in the vertical, z-axis direction, with polarization along the *x*-axis of the dimer (Figure S1B). In this Supplementary Information, the intensity of the electric field is reported for a different direction of polarization, that is, the *y* direction (Figure S1C). In this case, the enhancement of the electric field is vanishingly small, and this would prove that the device works as an effective SERS substrate.

Nanostructured substrates for SERS are expected to be anisotropic in terms of the local surface Plasmon resonances [7], which imply that the enhancement factor should extrinsically depend on the incident polarization. This effect is expected to be particularly strong for nanometric gaps between nanoparticles [8]. Here, we report on the polarization-dependent electrical field enhancement inside the gap from isolated silver dimmers studied by FDTD simulation.

Figure S1. The geometry of the simulated system (**A**) and the orientations of the incident radiation (**B**,**C**) utilized in the simulation of the EM field around the dimers**.**

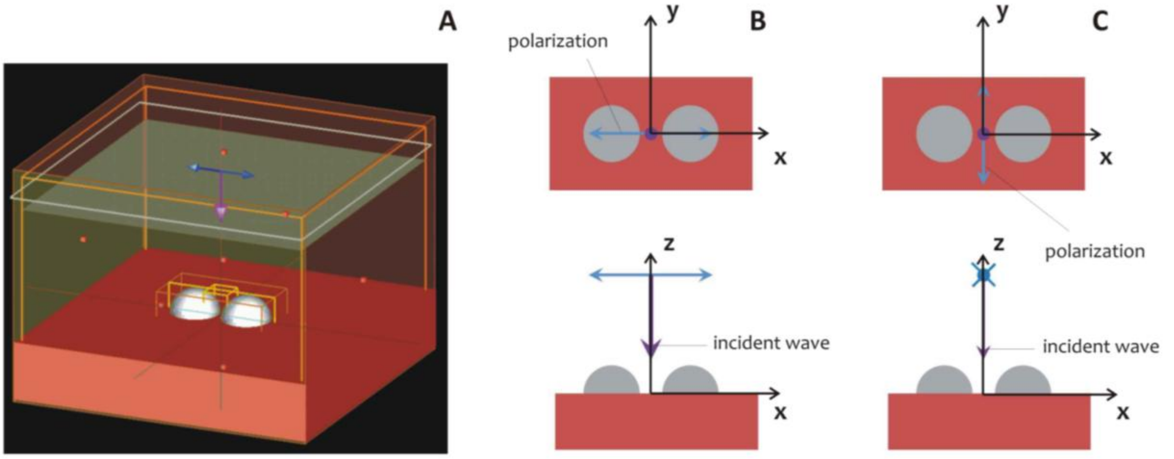

If we compare the results obtained in Figure 5 in the main text for a polarization parallel to the axis of the dimer with those reported in the Figure S2, where the polarization is perpendicular to this axis, a clear switch off effect is found. In the first case, in fact, is obtained an enhancement in the intensity of the electric field of the order of 10^3 while in the second is zeroed. In the Figure S2A,C there is an enhancement of 5 times for a share of around z = 33 nm which is attributed to the effects of reflection of the incident wave on the surface of the hemispheres. These results are found to be consistent with electrodynamics theory, confirming the plasmonic nature of the resonance observed in Figure 5 in the main text, and strongly support the idea that nano-gaps are a key ingredient of ultrasensitive SERS analysis. An explanation for this behavior can be given for small particles in which the electromagnetic interactions between the localized modes are essentially of a dipolar nature and so the particles can be treated as interacting dipoles. Using the simple approximation of interacting point dipoles, the direction of the resonance shifts for in-phase illumination can be determined by considering the Coulomb forces associated with the polarization of the particles. The restoring force acting on the oscillating electrons of each particle is either increased or decreased by the charge distribution of neighboring particles. Depending on the polarization direction of the exciting light, this leads to a blue-shift of the plasmon resonance for the excitation of transverse modes, and a red-shift for longitudinal modes.

Figure S2. The simulated EM field enhancement in along the *yz* (**A**) and *xz* (**B**) planes, and as a function of *z* (**C**) and wavelength (**D**).

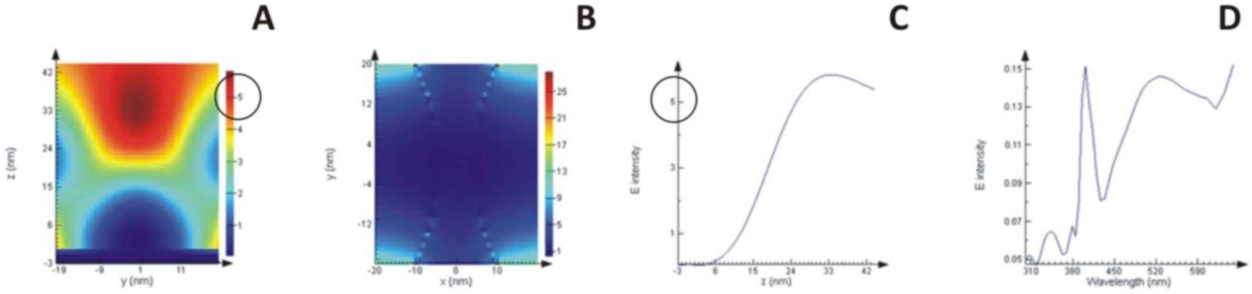

S5. Determining the Particle Size Distribution in A Pattern

While for the smaller pattern sizes, where often a single particle is present, the length or size of that particle could be measured directly, in the case of larger pattern sizes, where a large number of particles is deposited, an automatic image analysis algorithm was utilized for determining their size distribution.

The Matalab code is provided immediately after this discursive description. Also, you will find an example, that is, a cluster of nanoparticles in a pattern (Figure S3a), to which the algorithm has been applied to derive the granulometry (size distribution) of those nano-particles (Figure S3b).

Figure S3. SEM image of a nanoparticles cluster (**a**) and size distribution of particles in the image (**b**).

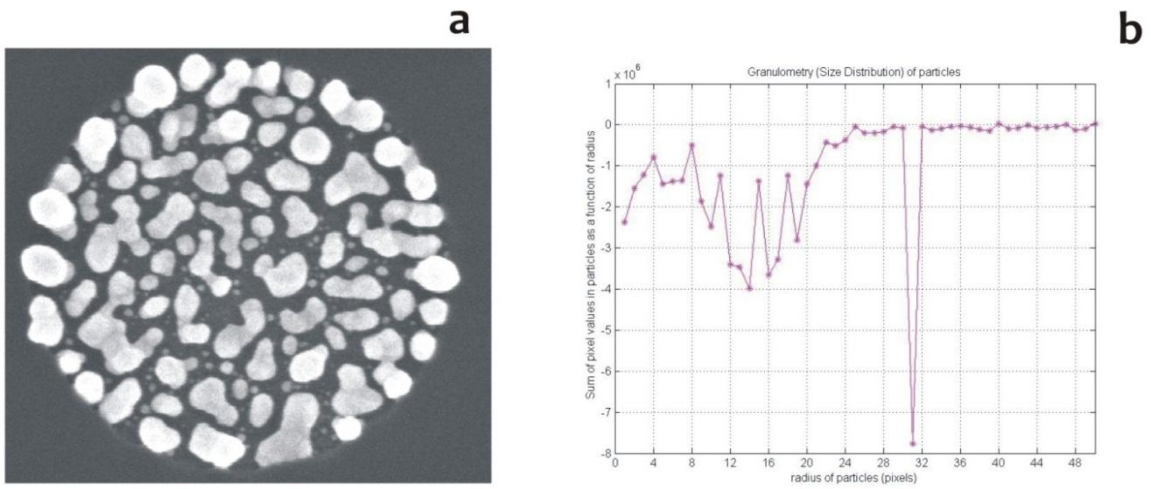

The algorithm determines the size distribution of objects in an image without explicitly segmenting (detecting) each object first. At the end, we will have the size distribution of single nanoparticles in a cluster of nanoparticles. In estimating the particle size distribution, a convenient conversion factor is utilized, whereby pixels in an image are converted in nanometers. This conversion factor is determined on measuring the size bar of an image in pixels and dividing it by its actual length in nano-meters. Therefore:

1. *The image interest is read an imported as a 16 bit graphic*.
2. *The intensity contrast in the image is enhanced*, performing contrast-limited adaptive histogram equalization. The image intensity is rescaled so that it fills the data type's entire dynamic range.
3. *The intensity surface area distribution in the enhanced image is determined*. The intensity surface area distribution of nano-particles as a function of size is determined. Image objects are compared to stones whose sizes can be determined by sifting them through screens of increasing size and collecting what remains after each pass. Image objects are sifted by opening the image with a structuring element of increasing size and counting the remaining intensity surface area (summation of pixel values in the image) after each opening.
4. *Calculate first derivative of distribution*. A significant drop in intensity surface area between two consecutive openings indicates that the image contains objects of comparable size to the smaller opening. This is equivalent to the first derivative of the intensity surface area array, which contains the size distribution of the particles in the image.

5. *Extract particles having a particular radius*. Notice the minima and the radii where they occur in the graph. The minima tell you that particles in the image have those radii. The more negative the minimum point, the higher the particles' cumulative intensity at that radius. In the example of Figure Sx.1b, the pronounced minimum at radius = 31 pixels indicate that 31 pixels is the size of the most frequent feature in the image. 31 pixels will be further converted in real dimensions using a convenient conversion factor as described above; for this particular configuration, 31 pixels correspond to a 110 nm diameter.

The original Matlab Code

```
function [n1]=sigprova(nome,psnm);
rgb = imread(nome);

I=rgb;

I = adapthisteq(I,'NumTiles',[1010]);
I = imadjust(I);
imshow(claheI);

for counter = 0:50
 remain = imopen(I, strel('disk', counter));
 intensity_area(counter + 1) = sum(remain(:));
end

figure,plot(intensity_area, 'm - *'), grid on;
title('Sum of pixel values in opened image as a function of radius');
xlabel('radius of opening (pixels)');
ylabel('pixel value sum of opened objects (intensity)');

imshow(remain);

intensity_area_prime= diff(intensity_area);

data=zeros(2);

data(1)=num;
data(2)=num2;

t = strcat(nome, '_sizeY.txt');
t2 = strcat(nome, '_sizeX.txt');

dlmwrite(t, intensity_area_prime, 'delimiter', '\t','precision', 5)
x = zeros(1,50);
for i = 1:50, x(i) = i; end
dlmwrite(t2, psnm*x, 'delimiter', '\t','precision', 5)

plot(intensity_area_prime, 'm - *'), grid on;
title('Granulometry (Size Distribution) of particles');
set(gca, 'xtick', [048121620242832364044485 2]);
xlabel('radius of particles (pixels)');
ylabel('Sum of pixel values in particles as a function of radius');
n1=3;
```

S6. Examples of SEM Images of the Nano-Grains at the Smallest Feature Size

We include here arrays of representative, ultra-high resolution SEM micrographs of the silver nano-grain aggregates at the smallest scales. The reader can now apprehend how those images have a sufficient degree of detail to allow to extracting useful information regarding the grain morphology even at the tiniest scales.

Figure S4. Array of ultrahigh resolution SEM images that demonstrate the capability of retrieving the particles size at the smallest nano-scales.

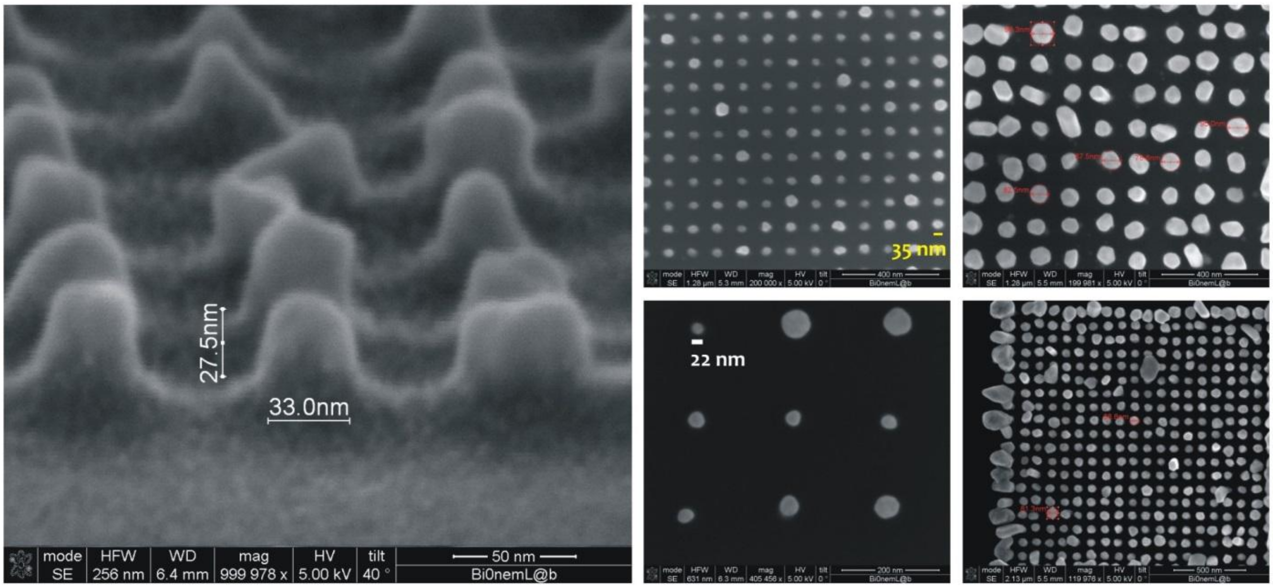

Figure S5. Array of ultrahigh resolution SEM images that demonstrate the capability of retrieving the particles size at the smallest nano-scales.

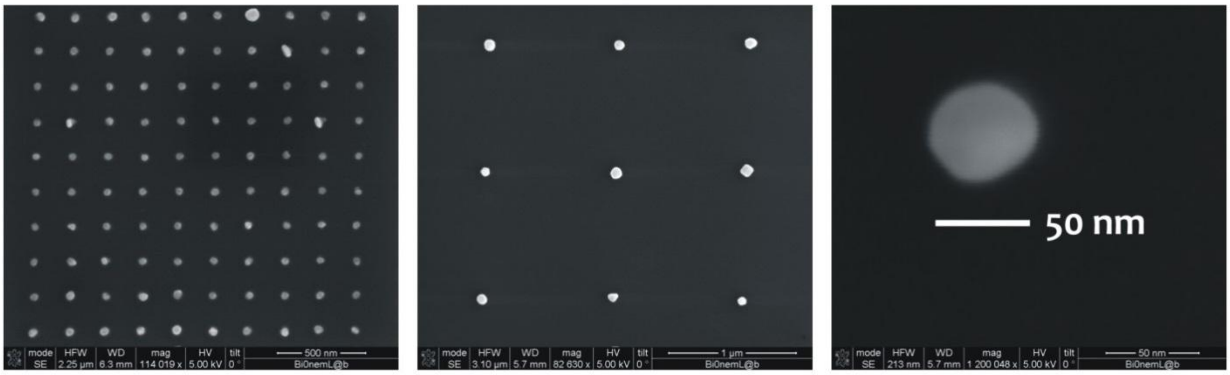

(**a**) (**b**) (**c**)

S7. Representative SEM Images of Clusters of Silver Nano-Grains with A Packing Factor Greater than One

In the particular case of $\delta > 1$, the cluster size is larger than the pattern diameter, as indicated by specific images reported in this section. In this case, the packing factor may be readily derived on measuring separately (a) the pattern size, that is the diameter of the exposed features in the EBL

sensitive resist prior electroless growth; and (b) the nano-grains size obtained upon exposition to the electroless solution, and calculating the ratio between the two.

Figure S6. Arrays of ultrahigh resolution SEM images show cases in which the packing factor of the structures is larger than one, that is, the diameter of the nanoparticles is greater than the pattern size.

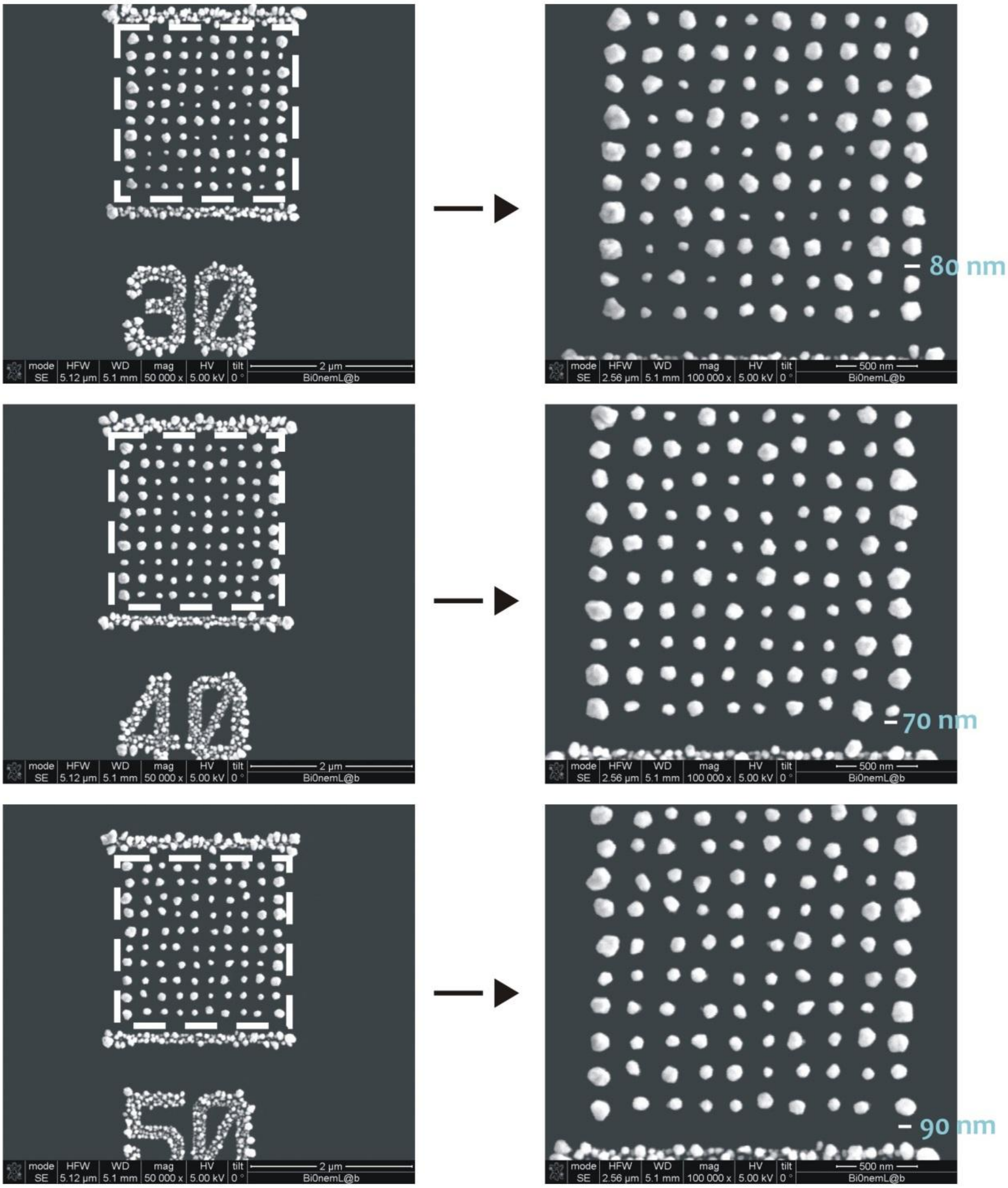

Figure S7. Arrays of ultrahigh resolution SEM images show cases in which the packing factor of the structures is larger than one, that is, the diameter of the nanoparticles is greater than the pattern size.

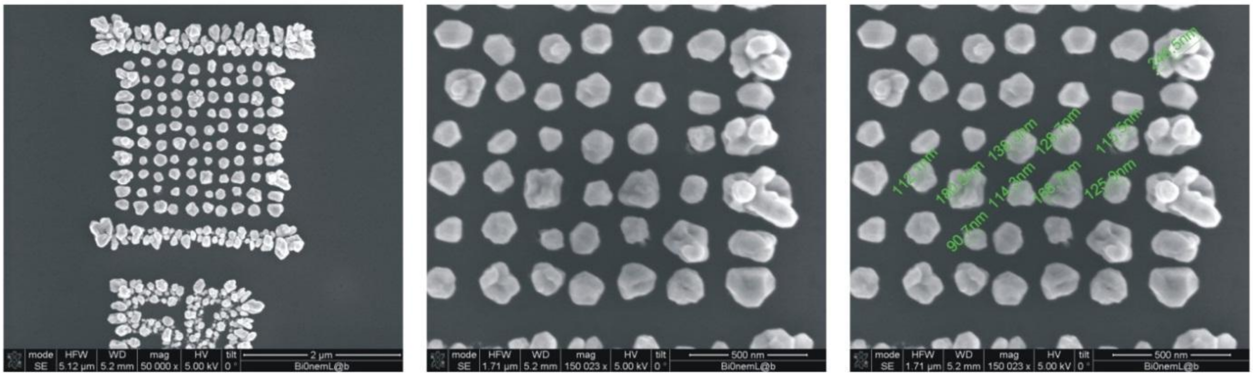

S8. SERS Controls on BT

Here we include additional data and Raman diagrams to substantiate the findings of the work. These are: (i) the reference spectrum of benzenethiol measured starting from a largely concentrated solution (10 mM), compared to the Raman signal of a diluted solution of benzenethiol (10^{-16} M) deposited upon a system of nanoparticle dimers (Figure S8A); (ii) the spectrum of benzenethiol (initial concentration 10^{-16} M) measured using a monomer particles array, compared to the Raman signal of the same solution acquired using a system of dimers (Figure S8B); (iii) the Raman signal of the solvent of benzenethiol alone (that is, ethanol), acquired upon a silver nanoparticle dimers SERS substrate (Figure S9).

On analyzing diagram (i), one can observe how the band centered at 1,580 cm^{-1}, that corresponds to the C–C stretching mode in the molecule of benzenthiol, can be realistically utilized as a reference band line for deriving the 3D Raman intensity map reported in Figure 5G in the main text.

On analyzing diagram (ii), one can notice how an array of silver nanoparticle dimers may enhance the Raman signal with a great efficiency in contrast to the modest (vanishingly small, for the considered concentration) enhancement of a simple system of particle monomers.

On analyzing diagram (iii), one can observe how the solvent evaporates completely upon the deposition on the substrate, this is particularly evident from the absence of any peak in correspondence of the frequency line centered at 3,300 cm^{-1}, that would correspond to the O–H stretching of the Ethanol molecule. Therefore, any signal measured on the substrate can be attributed to the BT. This would also confirm the absence of contaminants in solution.

Figure S8. Raman spectrum of BT measured in excess of solute in comparison to the SERS measurement of diluted BT on arrays of silver dimers (A), Raman spectrum of diluted BT measured on arrays of monomers of silver nanoparticles arrays compared to the Raman spectrum of BT measured on arrays of dimers of silver nanoparticles (B, C), .

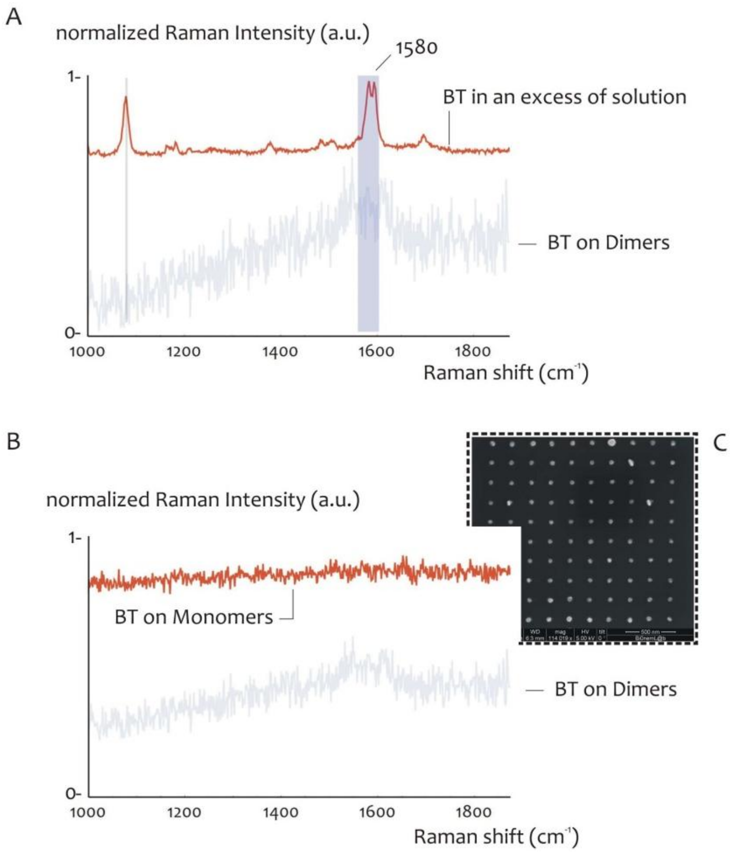

Figure S9. The Raman spectrum of BT measured on arrays of dimers of silver nanoparticles compared to the Raman spectrum of ethanol (that is, the solvent of BT), measured on the same substrate.

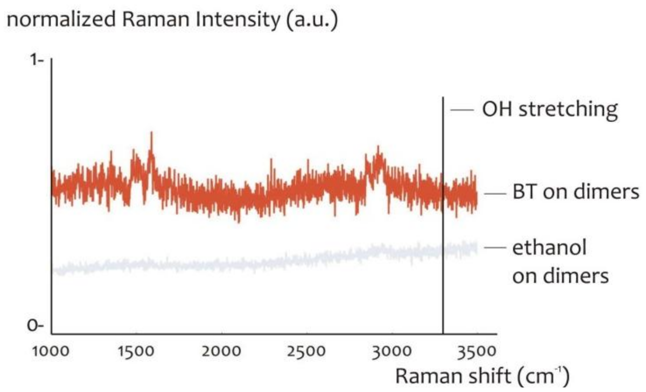

References

1. Haynes, W.M. *CRC Handbook of Chemistry and Physics*; CRC Press: Boulder, CO, USA, 1998.
2. Dechadilok, P.; Deen, W. Hindrance Factors for Diffusion and Convection in Pores. *Ind. Eng. Chem. Res.* **2006**, *45*, 6953–6959.
3. Saltzmann, M. *Drug Delivery*; Oxford University Press: London, UK, 2001.

4. Gentile, F.; Battista, E.; Accardo, A.; Coluccio, M.; Asande, M.; Perozziello, G.; Das, G.; Liberale, C.; De Angelis, F.; Candeloro, P.; *et al*. Fractal Structure Can Explain the Increased Hydrophobicity of NanoPorous Silicon Films. *Microelectron. Eng.* **2011**, *88*, 2537–2540.
5. Gentile, F.; Coluccio, M.; Toma, A.; Rondanina, E.; Leoncini, M.; De Angelis, F.; Das, G.; Dorigoni, C.; Candeloro, P.; Di Fabrizio, E. Electroless Deposition Dynamics of Silver Nanoparticles Clusters: A Diffusion Limited Aggregation (DLA) Approach. *Microelectron. Eng.* **2012**, *98*, 359–362.
6. Gentile, F.; Tirinato, L.; Battista, E.; Causa, F.; Liberale, C.; Di Fabrizio, E.; Decuzzi, P. Cells Preferentially Grow on Moderately Rough Substrates. *BioMaterials* **2010**, *31*, 7205–7212.
7. Kreibig, U.; Vollmer, M. *Optical Properties of Metal Clusters*; Springer: New York, NY, USA, 1995.
8. Xu, H.; Aizpurua, J.; Kall, M.; Apell, P. Electromagnetic contributions to single-molecule sensitivity in surface-enhanced Raman scattering. *Phys. Rev. E* **2000**, *62*, 4318–4324.

© 2014 by the authors; licensee MDPI, Basel, Switzerland. This article is an open access article distributed under the terms and conditions of the Creative Commons Attribution license (http://creativecommons.org/licenses/by/3.0/).
